# Altered lipid metabolism promoting cardiac fibrosis is mediated by CD34^+^ cell-derived FABP4^+^ fibroblasts

**DOI:** 10.1038/s12276-024-01309-9

**Published:** 2024-08-29

**Authors:** Luping Du, Xuyang Wang, Yan Guo, Tingting Tao, Hong Wu, Xiaodong Xu, Bohuan Zhang, Ting Chen, Qingbo Xu, Xiaogang Guo

**Affiliations:** 1 Department of Cardiology, The First Affiliated Hospital, School of Medicine, Zhejiang University, Hangzhou, China; 2 Department of Cardiovascular Surgery, First Affiliated Hospital, School of Medicine, Zhejiang University, Hangzhou, China; 3 Alibaba-Zhejiang University Joint Research Center of Future Digital Health care, Hangzhou, China

**Keywords:** Stem-cell differentiation, RNA sequencing, Stem-cell biotechnology

## Abstract

Hyperlipidemia and hypertension might play a role in cardiac fibrosis, in which a heterogeneous population of fibroblasts seems important. However, it is unknown whether CD34^+^ progenitor cells are involved in the pathogenesis of heart fibrosis. This study aimed to explore the mechanism of CD34^+^ cell differentiation in cardiac fibrosis during hyperlipidemia. Through the analysis of transcriptomes from 50,870 single cells extracted from mouse hearts and 76,851 single cells from human hearts, we have effectively demonstrated the evolving cellular landscape throughout cardiac fibrosis. Disturbances in lipid metabolism can accelerate the development of fibrosis. Through the integration of bone marrow transplantation models and lineage tracing, our study showed that hyperlipidemia can expedite the differentiation of non-bone marrow-derived CD34+ cells into fibroblasts, particularly FABP4^+^ fibroblasts, in response to angiotensin II. Interestingly, the partial depletion of CD34^+^ cells led to a notable reduction in triglycerides in the heart, mitigated fibrosis, and improved cardiac function. Furthermore, immunostaining of human heart tissue revealed colocalization of CD34^+^ cells and fibroblasts. Mechanistically, our investigation of single-cell RNA sequencing data through pseudotime analysis combined with in vitro cellular studies revealed the crucial role of the PPARγ/Akt/Gsk3β pathway in orchestrating the differentiation of CD34^+^ cells into FABP4^+^ fibroblasts. Through our study, we generated valuable insights into the cellular landscape of CD34^+^ cell-derived cells in the hypertrophic heart with hyperlipidemia, indicating that the differentiation of non-bone marrow-derived CD34^+^ cells into FABP4^+^ fibroblasts during this process accelerates lipid accumulation and promotes heart failure via the PPARγ/Akt/Gsk3β pathway.

## Hyperlipidaemia accelerates heart fibrosis via CD34+ cell differentiation

Cardiac fibrosis, a condition leading to heart failure, is caused by the activation of cardiac fibroblasts. These cells are influenced by various factors, including disorders in lipid metabolism. The role of lipid metabolism in cardiac fibrosis, particularly under conditions like hyperlipidemia and hypertension, is not fully understood. This study investigates how lipid metabolism disorders affect cardiac fibrosis and the role of certain cells in this process. The research uses human heart samples and mouse models, including a specific type of genetically modified mouse with induced hypertension. The study reveals that lipid metabolism disorders significantly contribute to cardiac fibrosis by promoting the transformation of CD34+ cells into FABP4+ fibroblasts, worsening heart fibrosis. The findings suggest potential new treatments for cardiac fibrosis.

This summary was initially drafted using artificial intelligence, then revised and fact-checked by the author.

## Introduction

Cardiac fibrosis is one of the main pathological processes in ventricular remodeling that leads to heart failure and is characterized by the activation of cardiac fibroblasts^1^. Due to the diverse heterogeneity and distinct subtypes of fibroblasts, these cells can influence myocardial fibrosis through diverse mechanisms. A range of cellular processes may contribute to this phenomenon, including resident fibroblast proliferation, endothelial mesenchymal transformation, and hematopoietic and bone marrow-derived cell differentiation^2^. However, the effect of lipid metabolism disorders on cardiac fibrosis has rarely been reported, and the relationships among these factors remain unclear.

The characteristics of fibroblasts and the precise origins (mature fibroblasts or stem/progenitor cells) of distinct fibroblast subtypes during cardiac fibrosis in the presence of lipid metabolism disorders and hypertension have not been definitively established.

Fatty acid-binding protein 4 (FABP4), also known as adipocyte FABP or aP2, represents a crucial subtype within the fatty acid-binding protein family^3^. It is expressed primarily in adipocytes and macrophages and plays an important role in the metabolically driven chronic inflammation observed in individuals with insulin resistance or atherosclerosis^4^. Recent studies have revealed that *FABP4* gene silencing or treatment with a specific FABP4 inhibitor (BMS309403) can attenuate lung and kidney interstitial fibrosis by modulating the macrophage-to-myofibroblast transition and the TGF-β pathway^5,6^. However, the precise function and underlying mechanism of FABP4 in fibroblast-mediated cardiac fibrosis are not well documented.

CD34+ cells are a functionally and originally heterogeneous population of cells that exert different effects on cardiovascular disease due to their diverse differentiation potential. In the last decade, it has been revealed that resident cells expressing CD34, c-Kit and Sca-1 lack the ability to differentiate into cardiomyocytes ^7–10^. However, in response to myocardial injury, these cells can differentiate into fibroblasts and endothelial cells^11,12^. Metabolic disorders are the drivers of cardiovascular disease. The influence of metabolic factors on CD34+ cells should not be ignored. However, it is still unclear whether CD34+ cells exhibit altered differentiation potential in the metabolic microenvironment (especially in lipid metabolism disorders) under conditions of hypertension and hyperlipidemia and whether CD34+ cells play a role in cardiac fibrosis. In addition, the function of CD34^+^ cells in heart failure caused by the combination of lipid overload and hypertension remains unknown. In our study, CD34-CreER;Rosa26-tdTomato&ApoE^-/-^ mice with angiotensin II-induced hypertension were used to observe the unique functions of CD34^+^ cells in hyperlipidemia and hypertension-induced cardiac remodeling through single-cell RNA sequencing (scRNA-seq) and genetic lineage tracing. Our results showed that a unique cluster of fibroblasts (FABP4^+^ fibroblasts) appeared in the presence of lipid overload and hypertension-induced cardiac remodeling. Resident CD34^+^ cells are the major cells that differentiate into FABP4^+^ fibroblasts, which exhibit the characteristics of disturbed lipid metabolism; CD34^+^ cell depletion in ApoE^-/-^ mice alleviated myocardial fibrosis, reduced triglyceride levels, and improved heart function when co-occurring with hypertension and dyslipidemia. Mechanistically, we demonstrated that the peroxisome proliferator activated receptor γ (PPARγ)/Akt/Gsk3β pathway was crucial for CD34+ cell-derived FABP4+ fibroblasts.

## Materials and methods

The detailed experimental methods are available in the **Supplementary Information**.

### Human heart samples

In this research, we analyzed three categories of left heart tissue samples obtained from humans. These categories consisted of three control samples (nonfailing, nontransplantable hearts) alongside two heart failure samples characterized by hyperlipidemia, as well as three heart failure samples presenting both hyperlipidemia and a history of hypertension. The control hearts were sourced from organ donors in good health, while the left ventricles of the heart failure patients were procured from patients undergoing heart transplantation. The heart failure patients were specifically diagnosed with dilated cardiomyopathy, exhibiting an ejection fraction (EF) below 30%. The study protocol received approval from the Medical Ethics Committee of the First Affiliated Hospital, Zhejiang University (Approval Reference No. 2021/330). Written informed consent was obtained from all patients, explicitly outlining the objective of our study, in accordance with the approved protocol.

### Mice

All animal experiments and protocols conducted in this study received approval from the Research Ethics Committees of the First Affiliated Hospital of Zhejiang University (Approval Reference No. 2021/159).

The following mouse strains were utilized: Cd34-CreERT2;Rosa26-tdTomato mice and Cd34-CreERT2; Rosa26-tdTomato-DTA (the mice were obtained as described previously^12^). Cd34-Dre knock-in mice (C57BL/6 background), Rosa26-RSR-LSL-tdTomato-2A (C57BL/6JSmoc-Gt(ROSA)26Sorem1(CAG-LSL-RSR-tdTomato-2A)Smoc) and Postn-CreERT2 knock-in mice, which were obtained from Shanghai Biomodel Organism Co. Cd34-CreERT2; Rosa26-tdTomato&ApoE^-/-^ mice were generated by crossing Cd34-CreERT2; Rosa26-tdTomato mice and ApoE^-/-^ mice. Cd34-CreERT2; Rosa26-tdTomato-DTA&ApoE^-/-^ mice were generated by crossing Cd34-CreERT2 mice, Rosa26-tdTomato-DTA mice, and ApoE^-/-^ mice. For dual-recombinase-activated lineage tracing, Cd34-Dre; Postn-CreER; tdTomato mice were produced by crossing Cd34-Dre, Postn-CreERT2, and Rosa26-RSR-LSL-tdTomato-2A-DTR mice with ApoE^-/-^ mice. Cd34-Dre; Postn-CreER; tdTomato&ApoE^-/-^ mice were generated by crossing Cd34-Dre; Postn-CreER; tdTomato mice with ApoE^-/-^ mice.

### Angiotensin II (Ang II)-induced cardiac hypertrophy model

The surgery was performed as described previously^13^. Adult male mice with an average weight of 25 ± 5 g were assigned randomly to various experimental groups. The specific number of mice used for each experiment is provided in the figure legends. Prior to experimentation, the animals were anesthetized using isoflurane, and the adequacy of anesthesia was continually assessed through the corneal and withdrawal reflexes. Additional details regarding the methods employed can be found in the Supplemental Materials and Methods section.

### CD34+ cell killing experiment

To eliminate CD34+ cells in mice, tamoxifen was administered orally via gavage for a duration of two weeks to CD34-CreER; Rosa26-tdTomato-DTA mice. Cardiac function was assessed using echocardiography four weeks after the surgical procedure.

### Bone marrow transplantation (BMT)

The BMT procedure was carried out following a previously described method^14^. The detailed methods are available in the Supplemental Materials and Methods.

### Isolating fibroblasts from the heart in mice and humans

The cell isolation methods were described previously. Following tissue dissociation, all procedures for cell culture experiments were meticulously conducted in a sterile environment to maintain sterility and prevent contamination. The detailed methods are available in the Supplemental Materials and Methods.

### Single-cell RNA sequencing

For the isolation of single cells from the mouse or human heart, a previously described protocol was followed^15^. The detailed methods are available in the Supplemental Materials and Methods. We performed scRNA-seq of six pooled samples at 0 and 4 weeks after surgery. For the scRNA-seq of noncardiomyocyte and tdTomato+ cells from the heart, standard Chromium™ Single Cell 3′ Reagent Kit v3 chemistry (10× Genomics) protocols were used. The procedure was carried out following a previously described method^12^.

### Gene set analysis

The gene set analyses, encompassing cell type genes and markers obtained from previously published studies and the PanglaoDB website, were conducted using the “AddModuleScore” function in Seurat^16,17^. The score of each cell was based on the expression levels of genes within each gene set in each cell.

### Mouse heart CD34+ ApoE^-/-^ cell isolation

The isolation of CD34-CreER;Rosa26-tdTomato ApoE^-/-^ mouse heart cells was conducted following established protocols. Subsequently, CD34+ cells were sorted using anti-CD34 magnetic beads following the instructions provided by the manufacturer. The detailed methods are available in the Supplemental Materials and Methods.

### Statistical analysis

GraphPad Prism 9.0 was used for generating the statistical graphs and conducting the comparative analyses. All the data are presented as the mean ± SEM. Prior to analysis, the normality distribution of the datasets was assessed using the Shapiro‒Wilk test. Subsequently, either Student’s *t*-test or one-way ANOVA followed by Tukey’s test was applied as appropriate. A p value less than 0.05 was considered to indicate statistical significance.

## Results

### Metabolic landscape of patients with hyperlipidemia and hypertension

**Fig. 1:**
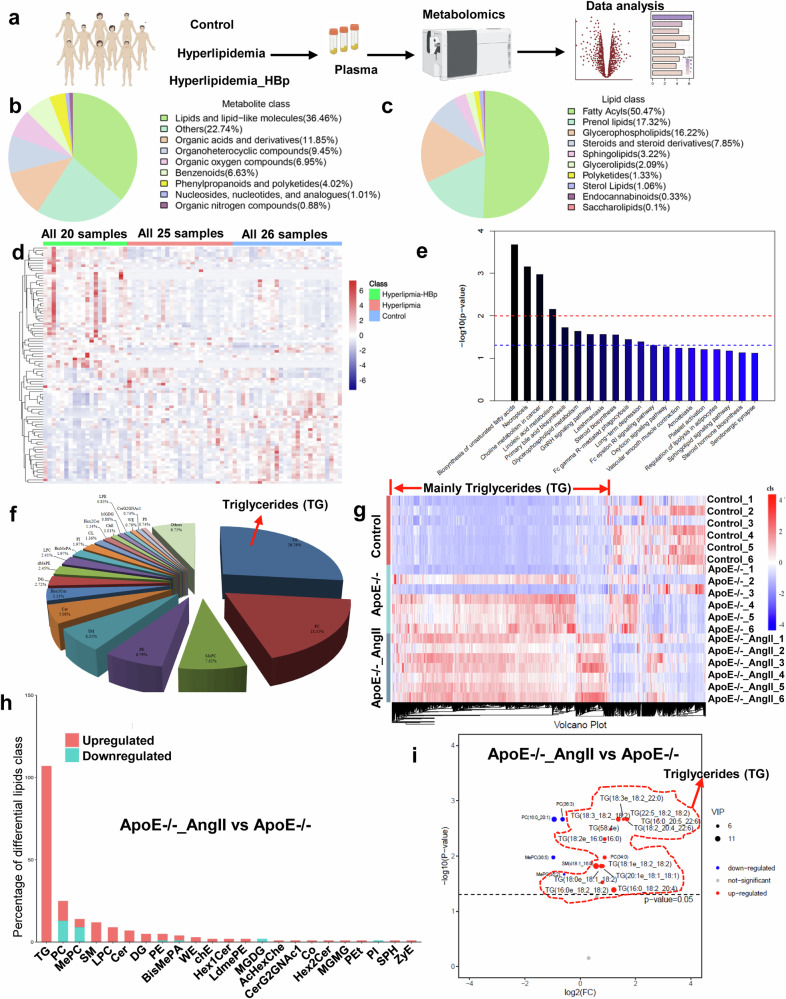
Metabolic landscape of patients with hyperlipidemia and hypertension. **a** Overview of the study design. **b**, **c** Counts and classes of metabolites detected by metabolomics in human serum. **d** The heatmap shows the different metabolites between the different groups. **e** KEGG analysis of differentially abundant metabolites. **f** Counts and classes of metabolites detected by metabolomics in mouse serum. **g** The heatmap shows the different metabolites between the different groups. **h** Bar chart of differential lipid classes. **i** Volcanic map of differential lipids.

To explore the overall metabolic changes in plasma samples obtained from patients with hyperlipidemia and hypertension, we performed untargeted metabolomic analyses on plasma collected from 25 patients with hyperlipidemia (hyperlipidemia group), 20 heart failure patients with hyperlipidemia and hypertension (hyperlipidemia_HBp group), and 26 normal individuals (control group) (Fig. 1a). The high quality of the human serum metabolomics was validated through the absence of batch effects and the consistent performance of the quality control (QC) samples (Supplementary Fig. 1a). In the three human groups, untargeted metabolomics detected 142 metabolites in the plasma, mainly lipids and lipid-like molecules, amino acids, nucleotides, organic acids, and acylcarnitines (Fig. 1b), all of which play crucial roles in cell growth and energy metabolism. In addition, lipid-related substances accounted for the majority of the metabolic substances (Fig. 1b). Fatty acids accounted for the majority of the lipid-related substances (Fig. 1c). However, we found that the metabolites in the different groups varied greatly; in particular, the hyperlipidemia_HBp group showed more changes in metabolic substances than did the hyperlipidemia group (Fig. 1d, Supplementary Fig. 1b). However, this method can only detect a very small number of triglycerides, as shown in Supplementary Fig. 1d. Compared with those in the hyperlipidemic group, the individual metabolites in the fatty acid or triglyceride group (red box) differed greatly between the hypertension and hyperlipidemic groups (Supplementary Fig. 1d).

We then performed KEGG analysis on these different metabolites and found that most metabolites were related to lipid metabolism pathways (such as biosynthesis of unsaturated fatty acids, regulation of lipolysis in adipocytes, glycerophospholipid metabolism, and linoleic acid metabolism) (Fig. 1e). As shown in Supplementary Fig. 1c, the fatty acid transporter family (aFABP) and PI3K/Akt pathways were involved. These findings demonstrate that lipid metabolism plays a significant role in hyperlipidemia, hyperlipidemia and hypertension.

We performed lipidomic analyses on plasma collected from mice (control, hyperlipidemia [ApoE^-/-^ group], heart failure with hyperlipidemia, and hypertension [ApoE^-/-^ with angiotensin II group]). The high quality of the mouse serum lipid metabolomics data was validated through the absence of batch effects and the consistent performance of quality control (QC) samples (Supplementary Fig. 2a). As shown in Fig. 1f, triglycerides (TGs) comprised the majority of the lipids (Fig. 1f). Moreover, we found that the lipid metabolites in the different groups varied greatly; moreover, triglycerides were the most varied among them (Fig. 1g, Supplementary Fig. 2b–d, g). In particular, the angiotensin II group showed more changes in TG than did the ApoE^-/-^ group (Fig.1h, i, Supplementary Fig. 2e). Moreover, as shown in Supplementary Fig. 2f, we also found that triglycerides accounted for the majority of the differential lipid associations between the different treatment groups and were significantly associated with other lipids (Supplementary Fig. 2f). This suggests that dynamic changes in lipid metabolites occurred within the three groups and that the changes in TG were the most significant when hypertension was combined with hyperlipidemia.

### ScRNA-seq analysis of noncardiomyocytes from heart failure patients with hyperlipidemia and hypertension

Increasing evidence suggests that noncardiomyocytes play pivotal roles in the pathogenesis of cardiac diseases^12,13,18,19^. It has also been confirmed that metabolic remodeling is an early event in the progression of diseases, and the heart can remodel its metabolic pathways during chronic pathophysiological conditions, leading to alterations in myocardial energetics and contractile function^20^. Therefore, our goal was to elucidate the dynamic changes occurring in noncardiomyocyte cells during heart failure in different metabolic states.

**Fig. 2:**
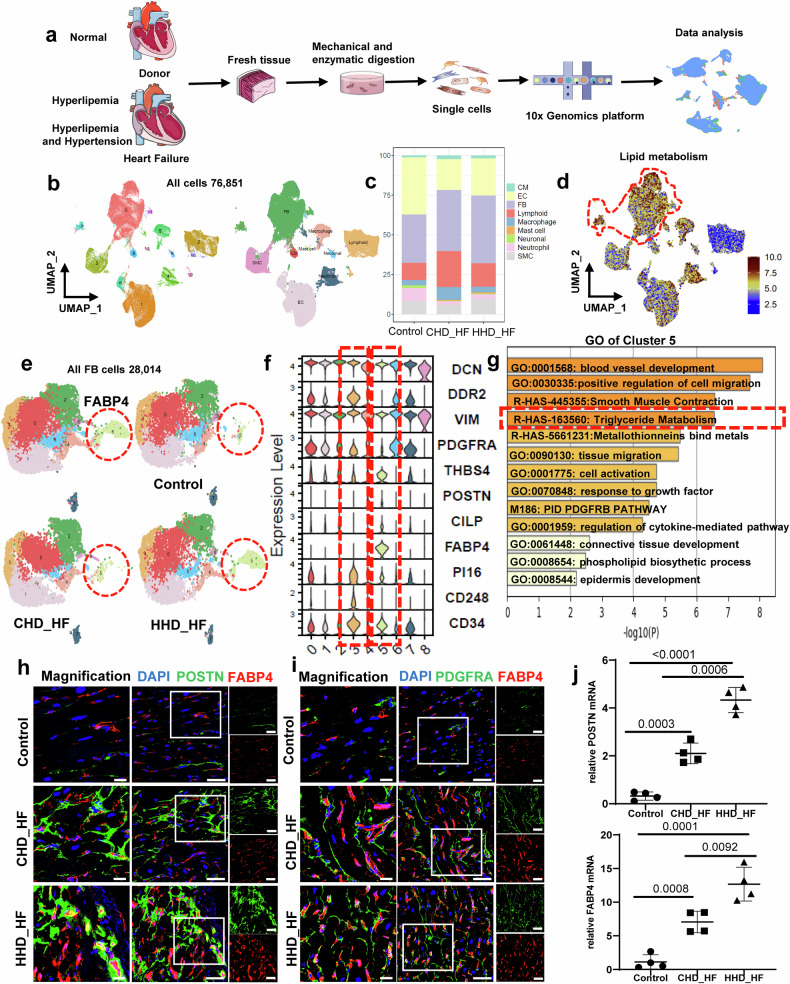
ScRNA-seq analysis of noncardiomyocytes from heart failure patients with hyperlipidemia and hypertension. **a** Schematic depicting the pipeline of human heart and tissue harvesting for scRNA-seq. Single cells were then isolated from the left ventricle and subjected to scRNA-seq. **b** UMAP plot displaying the major cell types and color-coded cell clusters of human hearts. **c** Bar chart showing the percentages of major cell types among different datasets. **d** UMAP plot displaying the distribution of lipids according to GSVA. **e** UMAP plot displaying the distribution of fibroblast subpopulations. Resolution = 0.5. **f** Violin plot showing the expression of selected marker genes in each subcluster. **g** GO analysis of Cluster 5 showed that FABP4+ fibroblasts were enriched in the triglyceride metabolism pathway (R-HAS-163560, circled by the red rectangle). **h, i** Representative images showing specific cells identified by staining for POSTN, FABP4, and PDGFRa. Scale bars, 50 μm and 20 μm in magnified images. **j** mRNA levels of a fibroblastic marker (Postn) and FABP4 in total fibroblasts isolated from patient hearts. GAPDH was used as an internal control, *n* = 4. The data are given as mean ± SEM. *P* < 0.05 was considered to indicate statistical significance.

We acquired single-cell transcriptomes from 76,851 cells by integrating three groups derived from human hearts (control: normal heart; CHD_HF: heart failure with a history of coronary heart disease; and HHD_HF, heart failure with a history of coronary heart disease and hypertension) after strict quality control (Fig. 2a, b). Utilizing their distinctive molecular signatures, we classified nine major cell types, comprising fibroblasts (Fibroblasts, DCN^+^, DDR2^+^), cardiomyocytes (CM, ACTA1^+^), endothelial cells (EC, VWF^+^), macrophages (CD68^+^), neuronal cells (PLP1^+^), lymphoid cells (IL7R^+^, CD79A^+^), neutrophils (S100A8^+^), smooth muscle cells (SMC, MYH11^+^) and mast cells (KIT^+^) (Fig. 2a, Supplementary Fig. 3a–c). As shown in Fig. 2c, in the CHD-HF group, the proportion of fibroblasts was notably greater than that in the control group. More importantly, compared to that in the CHD_HF group, the proportion of fibroblasts in the HHD_HF group was greater (Fig. 2c).

Heatmap analysis revealed distinct metabolic transcriptome signatures among the various groups. As shown in Supplementary Fig. 3d, the metabolic gene expression signatures differed among the different groups (Supplementary Fig. 3d), as did the noncardiomyocytes between the different groups (Fig. 2b, c), possibly reflecting that the changes in metabolic status may have had an effect on the noncardiomyocytes. Considering that lipid metabolites accounted for the majority of the metabolic substances, we focused on the effects of the changes in the lipids on noncardiomyocytes (especially fibroblasts). Interestingly, the UMAP plot of gene set variation analysis (GSVA) showed that the metabolic transcriptome signatures related to lipid metabolism were mainly related to fibroblasts (Fig. 2d). Fibroblasts play a vital role in myocardial fibrosis^21,22^. Therefore, in subsequent experiments, fibroblasts were extracted from all groups and partitioned into distinct subtypes. The UMAP plots revealed a notable dispersion of fibroblast clusters, indicating a considerable degree of fibroblast heterogeneity (Fig. 2e). Particularly noteworthy was the significant alteration in the percentage of several distinct fibroblast subtypes in the heart failure groups compared to the control group (Fig. 2e, Supplementary Fig. 4b). Analysis of cell abundance within this single-cell RNA sequencing dataset revealed several cell populations with varying relative abundances among the groups. In line with the results of previous reports^12^, the CHD_HF and HHD_HF groups exhibited a significantly greater proportion of fibroblasts than the control group (Fig. 2c, Supplementary Fig. 4b). In addition, as shown in the volcano plot, the levels of active fibroblast markers (POSTN, THBS4, CILP, and FN1) were increased in the heart failure group (Supplementary Fig. 4c).

In our datasets, we identified a specific subgroup (Cluster 5, termed FABP4^+^ fibroblasts) that specifically expressed FABP4 at high levels (Fig. 2e, f, Supplementary Fig. 4a, e). To our surprise, Gene Ontology (GO) enrichment analysis of FABP4^+^ fibroblasts revealed that this gene was associated with TG metabolism (Fig. 2g); in addition, GSVA of the fibroblast subgroups demonstrated that the genes related to lipid metabolism were mainly concentrated in this FABP4^+^ fibroblast subgroup (Supplementary Fig. 4f). As shown in Supplementary Fig. 4b and 2e, compared to that in the CHD_HF and control groups, FABP4^+^ fibroblast expression changed the most significantly in the HHD_HF group (Fig. 2e), and FABP4 expression in the HHD_HF group was significantly greater than that in the CHD_HF group (Fig. 2e). These data indicate that the change in FABP4^+^ fibroblasts was the most significant in the context of hypertension combined with hyperlipidemia. In addition, immunofluorescence staining demonstrated elevated expression levels of fibroblast markers (such as POSTN and PDGFRA) and FABP4+ fibroblasts (FABP4 + POSTN+ and FABP4 + PDGFRA+) in the heart failure group compared to those in the control group. Furthermore, these levels were notably increased in comparison to those in the CHD group (Fig. 2h, i and Supplementary Fig. 4i). Moreover, by examining the expression levels of fibroblasts (including POSTN, VIMENTIN and DDR2) and FABP4 in total fibroblasts isolated from the patient’s heart, we observed elevated expression levels of fibroblast markers, including POSTN, VIMENTIN, DDR2, and FABP4, in the heart failure group compared to the control group (Fig. 2j and Supplementary Fig. 4d). Additionally, these expression levels were significantly higher than those in the CHD_HF group (Fig. 2j and Supplementary Fig. 4d). Together, these data suggest that hypertension combined with hyperlipidemia can further accelerate myocardial fibrosis compared to hyperlipidemia alone, especially in the FABP4^+^ fibroblast subcluster.

Surprisingly, Cluster 3 exhibited increased expression of progenitor cell markers, including CD34, PI16, and CD248, which are involved in multilineage differentiation^12,23–25^ (Fig. 2f), further suggesting that Cd34-high fibroblasts might perform distinct functions during cardiac homeostasis and in response to injury. Hence, we examined the potential developmental relationships among these fibroblast subclusters. Pseudotime analysis was performed to visualize the trajectories of fibroblast differentiation across the steady-state atlas, with Cluster 3 designated as the root. This allowed us to order the fibroblasts along a trajectory, revealing distinct cell states (Fig. 2e, f and Supplementary Fig. 4e, f). Notably, the expression patterns of CD34, PI16, and FABP4 exhibited significant changes in accordance with the identified pseudotime trajectory (Supplementary Fig. 4g).

### 3.3 scRNA-seq analysis of noncardiomyocytes from ApoE^-/-^ mice with hyperlipidemia and hypertension

**Fig. 3:**
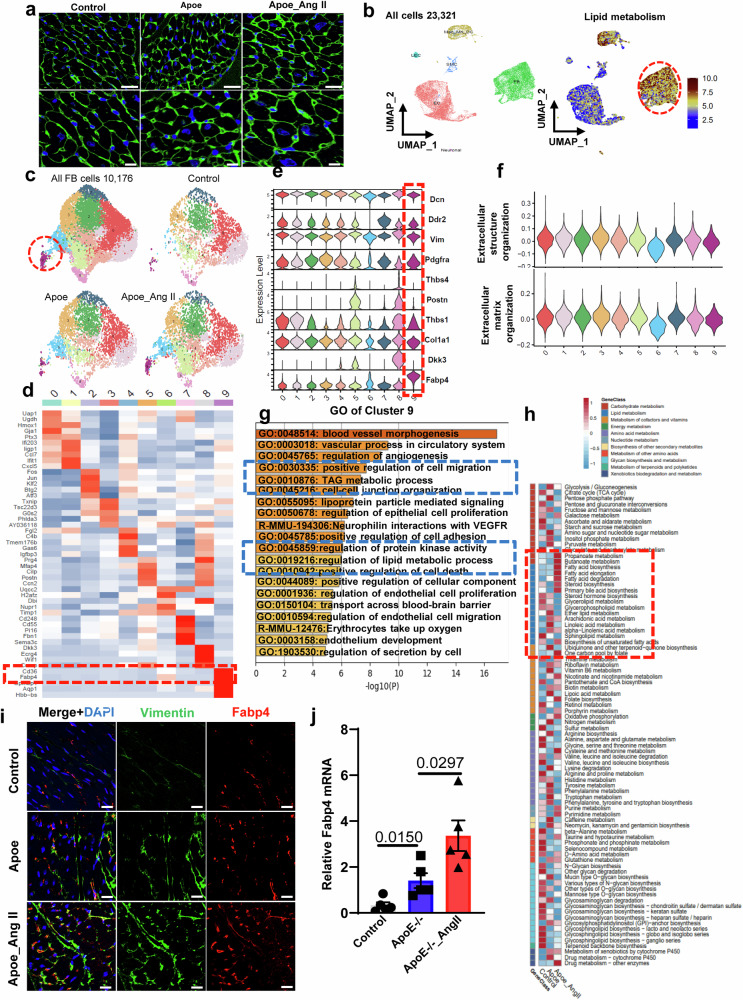
scRNA-seq analysis of noncardiomyocytes from ApoE^-/-^ mice with hyperlipidemia and hypertension. **a** Wheat germ agglutinin (WGA) staining showing cardiomyocyte cell size. Scale bars, 50 μm and 20 μm in magnified images. **b**. UMAP plot displaying the major cell types and color-coded cell clusters of mouse hearts; UMAP plot displaying the distribution of lipids by GSVA. **c** UMAP plot displaying the distribution of fibroblast subpopulations among different datasets after Ang II surgery. Resolution = 0.5. **d** Heatmap showing the expression of the top ten differentially expressed genes in each cell subcluster. **e** Violin plot showing the expression of selected marker genes in each subcluster. **f** Violin plot showing the GO dataset scores among fibroblast subclusters. **g** Bar plot showing the GO enrichment of selected subclusters. **h** GSVA of fibroblasts from the three groups. **i**. Representative images showing specific cell identification by staining for Vimentin and Fabp4. Scale bars, 20 μm. **j** mRNA levels of Fabp4 in total fibroblasts isolated from mouse heart; GAPDH was used as an internal control. Data are given as the mean ± SEM, *n* = 5. *P* < 0.05 was considered to indicate statistical significance.

As shown in Supplementary Fig. 4h, by analyzing the correlation between human and mouse single-cell datasets, we found that the cellular compositions of mouse and human hearts were very similar (Supplementary Fig. 4h). To gain a more in-depth understanding of the precise roles of CD34+ cells in cardiac fibrosis after injury and to explore the relationship between CD34+ cells and FABP4+ fibroblasts in vivo, we employed an inducible lineage-tracing knock-in mouse model (*Cd34*-CreERT2;Rosa26-tdTomato&ApoE^-/-^). To understand the pathology of progressive heart failure, we established a model of angiotensin II-induced hypertrophy, which included control, ApoE^-/-^, and ApoE^-/-^ mice with hypertrophy and a reduced ejection fraction (ApoE^-/-^angiotensin II group, early heart failure). We conducted echocardiographic imaging to assess the cardiac function of the two distinct genotypes of mice by measuring parameters such as the left ventricle ejection fraction (EF) and fractional shortening (FS). The data indicated that angiotensin II administration significantly reduced cardiac function compared to that in the sham group (Supplementary Fig. 5a). Compared to those in the group without angiotensin II, the blood pressure of the mice in the angiotensin II group was significantly increased (Supplementary Fig. 5a). Cardiac tissues were collected after angiotensin II injury, and wheat germ agglutinin (WGA) staining revealed the cardiomyocyte cell size (Fig. 3a). In addition, we found a noteworthy increase in blood pressure in the AngII-treated mice (Supplementary Fig. 5b). Additionally, there was also a significant increase in lipids (triglycerides and total cholesterol) in the ApoE^-/-^ and ApoE^-/-^_AngII groups compared to those in the control group (Supplementary Fig. 5c). These outcomes verified the successful establishment of the mouse model.

To unravel the cellular landscape of noncardiomyocytes in response to hyperlipidemia and hypertension, we obtained 23,321 single cells by integrating three groups from mouse hearts (control, ApoE^-/-^, ApoE^-/-^_Ang II) after strict quality control and defined six major cell types, which included fibroblasts (Dcn^+^, Ddr2^+^), EC (Cdh5^+^), neuronal cells (Plp1^+^), Mac_MO_DC cells (Cd68^+^), and SMC (Acta2^+^), based on their respective molecular signatures (Fig. 3b, Supplementary Fig. 5d–g). As shown in Supplementary Fig. 6a, b, GSVA revealed that different groups exhibited different metabolic transcriptome signatures along with different metabolic gene expression signatures among the different cell types (Supplementary Fig. 6a, b), possibly reflecting that the changes in metabolic status may have had an effect on noncardiomyocytes in the mouse model.

Interestingly, GSVA revealed that the metabolic transcriptome signatures related to lipid metabolism were mainly concentrated in fibroblasts (Fig. 3b). Among the noncardiomyocytes, fibroblasts are the predominant cell type and serve as the primary effector cells during myocardial fibrosis^18,26,27^. Therefore, in subsequent experiments, fibroblasts were extracted and classified into distinct subtypes from all three groups. The UMAP plots and the bar chart revealed a considerable degree of heterogeneity among the fibroblast clusters (Fig. 3c, Supplementary Fig. 5h). GO enrichment analysis revealed an elevated capacity for extracellular matrix (ECM) organization, extracellular structure organization, and ossification among all of the fibroblast clusters involved in fibrosis development (Fig. 3f).

Similar to previous results in human cells, we found a group of fibroblast subsets with high expression of FABP4 (Cluster 9, termed FABP4^+^ fibroblasts) (Fig. 3e), and GO enrichment analysis revealed that FABP4-high fibroblasts exhibited significant enrichment in TG metabolic processes and the regulation of lipid metabolic processes (Fig. 3g). In addition, GSVA of fibroblasts from the three groups revealed that fibroblasts in the ApoE^-/-^ group and angiotensin II group had significantly greater lipid metabolism than did those in the control group. Furthermore, compared with those in the ApoE^-/-^ group, the fibroblasts in the angiotensin II group exhibited more pronounced changes in fatty acid biosynthesis, fatty acid elongation, fatty acid degradation, and unsaturated fatty acid biosynthesis (Fig. 3h and Supplementary Fig. 6b).

Surprisingly, in contrast to those in the control group, there was a notable increase in the number of fibroblasts (Vimentin, Ddr2, and POSTN) in the ApoE^-/-^ group (Fig. 3j, Supplementary Fig. 6d). In addition, compared to those in the ApoE^-/-^ group, the number of fibroblasts in the angiotensin II group was significantly increased (Fig. 3i, j, Supplementary Fig. 6c, d). Furthermore, we found that the abundances of FABP4^+^Vimentin^+^, FABP4^+^PDGFRα^+^, and FABP4^+^FAP^+^ cells were significantly greater in the angiotensin II group than in the ApoE^-/-^ group (Fig. 3i, Supplementary Fig. 6c and h). In the total fibroblasts isolated from the mouse heart, we found that the expression levels of fibroblast markers, such as Postn, Vimentin, Ddr2, and Fabp4, were elevated in the ApoE^-/-^ group compared to those in the control group and further significantly increased in the angiotensin II group compared to those in the ApoE^-/-^ group (Fig. 3j and Supplementary Fig. 6d). These results indicate that the disruption of lipid metabolism accelerates the production of fibroblasts, which is significantly increased when combined with hypertension. Similar to the human data, we also found that one subset of fibroblasts (Cluster 7) displayed heightened expression of several progenitor cell markers, such as Cd34, Ly6a, Pi16, and Thy1^18^, in mice (Fig. 3d), suggesting that Cd34-high fibroblasts may have distinct functions in myocardial fibrosis.

**Fig. 4:**
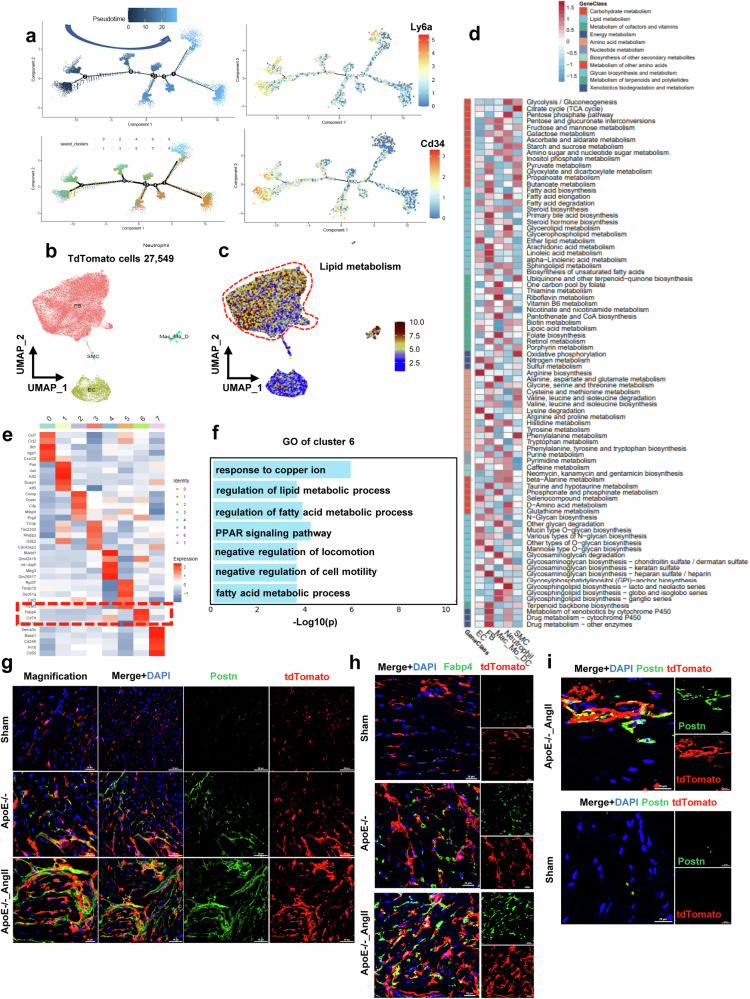
Lineage tracing study and scRNA-seq analysis of CD34+ cells in ApoE^-/-^ mice with hyperlipidemia and hypertension. **a** Pseudotime-dependent expression of Cd34 and Ly6a. **b** UMAP plot displaying the major cell types and color-coded cell clusters at different stages of pathological cardiac hypertrophy. **c**. UMAP plot displaying the distribution of lipids according to GSVA. **d** GSVA of all cell types. **e** Heatmap showing the expression of the top ten DEGs in each cell subcluster. **f** Bar plot showing the GO enrichment of selected subcluster 6. **g** Representative images showing specific cell identification by staining for Postn and tdTomato. **h** Representative images showing specific cell identification by staining for Fabp4 and tdTomato. **i** Representative images showing specific cell identification by staining for Postn and tdTomato in dual-recombinase-activated lineage tracing mice (Cd34-Dre;Postn-CreER;tdTomato&ApoE^-/-^).

Therefore, to explore the potential developmental relationships among these fibroblast subclusters, we visualized the pseudotime(s) using principal curves, which represent the trajectories of fibroblast differentiation across the steady-state atlas. For this analysis, Cluster 7 was designated as the root. This allowed us to order the fibroblasts along a trajectory and identify distinct cell states during the process (Fig. 4a, Supplementary Fig. 6e–g). The expression of Cd34, Pi16, and Ly6a significantly changed with the state, corresponding to the pseudotime trajectory (Fig. 4a, Supplementary Fig. 6e–g). Combining the human and mouse data, we concluded that a subpopulation of CD34+ cells in the heart exhibits a progenitor cell-like phenotype capable of transdifferentiating into various cell types, particularly fibroblasts, and this specific role in cardiac fibrosis highlights their significance in the pathological process.

### Lineage tracing and scRNA-seq analysis of CD34^+^ cells in ApoE^-/-^ mice

To better explore the specific functions of CD34^+^ cells in cardiac fibrosis after injury and the relationship between CD34^+^ cells and FABP4+ fibroblasts in vivo, we employed an inducible lineage tracing knock-in in a mouse model (*Cd34*-CreERT2;Rosa26-tdTomato&ApoE^-/-^). During the experiment, mice received five consecutive pulses of tamoxifen treatment over the course of one week to induce tdTomato labeling specifically in CD34+ cells. To confirm the successful induction by tamoxifen, Cd34-CreERT2; Rosa26-tdTomato&ApoE^-/-^ mice were examined using an in vivo imaging system both with and without tamoxifen induction (Supplementary Fig. 7a).

To comprehensively map the distribution of cardiac-resident CD34-lineage cells in patients with cardiac hypertrophy with hyperlipidemia, we conducted scRNA-seq on CD34-lineage cells (tdTomato+ cells). After rigorous quality control, a total of 27,549 single cells were available for subsequent analysis (Figs. 4b, 7b). According to their transcriptome characteristics, the cells were classified into six major cell types: fibroblasts (fibroblasts, Dcn+, Ddr2+), Mac_MO_DC (Cd68+), endothelial cells (EC, Cdh5+) and smooth muscle cells (SMC, Acta2+). (Fig. 4b, Supplementary Fig. 7b–e).

Interestingly, GSVA of the scRNA-seq dataset of CD34 lineage cells also revealed that the metabolic transcriptome signatures related to lipid metabolism were mainly concentrated in fibroblasts (Fig. 4c, d). Subsequently, we focused on CD34-lineage fibroblasts to uncover the heterogeneous features among the three groups. The extracted fibroblast population was further subdivided into eight distinct subclusters (Supplementary Fig. 7f). Compared to those in the ApoE^-/-^ group, the fibroblasts in the angiotensin II group exhibited significantly greater fatty acid metabolism (Supplementary Fig. 8a, b). Similar to previous human and mouse results, we found a group of fibroblast subsets with high FABP4 expression (Cluster 6) (Fig. 4e, Supplementary Fig. 7g). Furthermore, GO enrichment analysis revealed that the regulation of lipid metabolic processes, fatty acid metabolic processes, fatty acid metabolic processes, and the PPAR signaling pathway were highly enriched in Cluster 6 (FABP4^+^ fibroblasts) (Fig. 4f).

Intriguingly, there was a significant increase in the number of CD34-lineage fibroblasts (Vimentin, Ddr2, and POSTN) in the ApoE^-/-^ group compared to the control group. The number of CD34-lineage fibroblasts in the angiotensin II group was significantly greater than that in the ApoE^-/-^ group (Fig. 4g, Supplementary Fig. 8c, d). Furthermore, the percentage of CD34-lineage fibroblasts (PDGFRa+tdTomato+) in heart tissues was also greater in the ApoE^-/-^ group than in the control group according to flow cytometry analysis (Supplementary Fig. 8h). The percentage of CD34-lineage fibroblasts (PDGFRa+tdTomato+) was also significantly greater than that in the ApoE^-/-^ group according to flow cytometry analysis (Supplementary Fig. 8h). In addition, the number of CD34-expressing FABP4-positive fibroblasts was significantly greater in the angiotensin II group than in the ApoE^-/-^ group (Fig. 4h). These data suggest that disturbances in lipid metabolism can accelerate the differentiation of CD34^+^ cells into fibroblasts (especially FABP4^+^ fibroblasts), which are significantly increased in patients with hypertension.

To further clarify the differentiation of CD34^+^ cells into fibroblasts rather than the proliferation of CD34^+^ cells in cardiac fibrosis, we successfully constructed CD34Dre, PostnCreERT2, and R26-RSR-LSL-TdT (Cd34-Dre; Postn-CreER;tdTomato) dual-recombinase-activated lineage-tracing mice (Supplementary Fig. 8e). For this dual-recombinase-activated lineage tracing, the mouse was labeled with tdTomato fluorescence only if the offspring cells were derived from CD34 and expressed Postn. Cd34-Dre, Postn-CreER, and tdTomato&ApoE^-/-^ mice were generated by crossing Cd34-Dre, Postn-CreER, tdTomato, and ApoE^-/--/-^ mice. The mice were then treated with angiotensin II (Supplementary Fig. 8f). As shown in Fig. 4i, in the area of myocardial fibrosis, we found large numbers of tdTomato^+^ cells and Postn^+^ cells but only a small number of CD34^+^ cells after angiotensin II treatment compared to those in the sham group (Fig. 4i). Furthermore, tdtomato^+^Postn^+^ cells accounted for more than 90% of the Postn^+^ cells (Supplementary Fig. 8g). These results revealed that fibroblasts are mainly derived from the differentiation of CD34^+^ cells rather than from their proliferation during myocardial fibrosis. This further indicates that CD34^+^ cells have a progenitor cell-like phenotype.

### ScRNA-seq analysis revealed the role of lipid metabolism disturbance in myocardial fibrosis

To further clarify the role of hyperlipidemia in hypertrophy-induced fibrosis, we performed scRNA-seq analysis of Cd34-CreERT2; Rosa26-tdTomato&ApoE^-/-^ and Cd34-CreERT2; Rosa26-tdTomato mice after surgery. Transverse aortic constriction (TAC) surgery and angiotensin II are the most commonly used methods to induce myocardial hypertrophy, both of which can lead to myocardial hypertrophy by affecting cardiac afterload. Thus, they have similar effects to some extent. Therefore, we performed scRNA-seq analysis of Cd34-CreERT2, Rosa26-tdTomato&ApoE^-/-^ (Angiotensin II 4w), Cd34-CreERT2, and Rosa26-tdTomato (TAC 4W) mice.

**Fig. 5:**
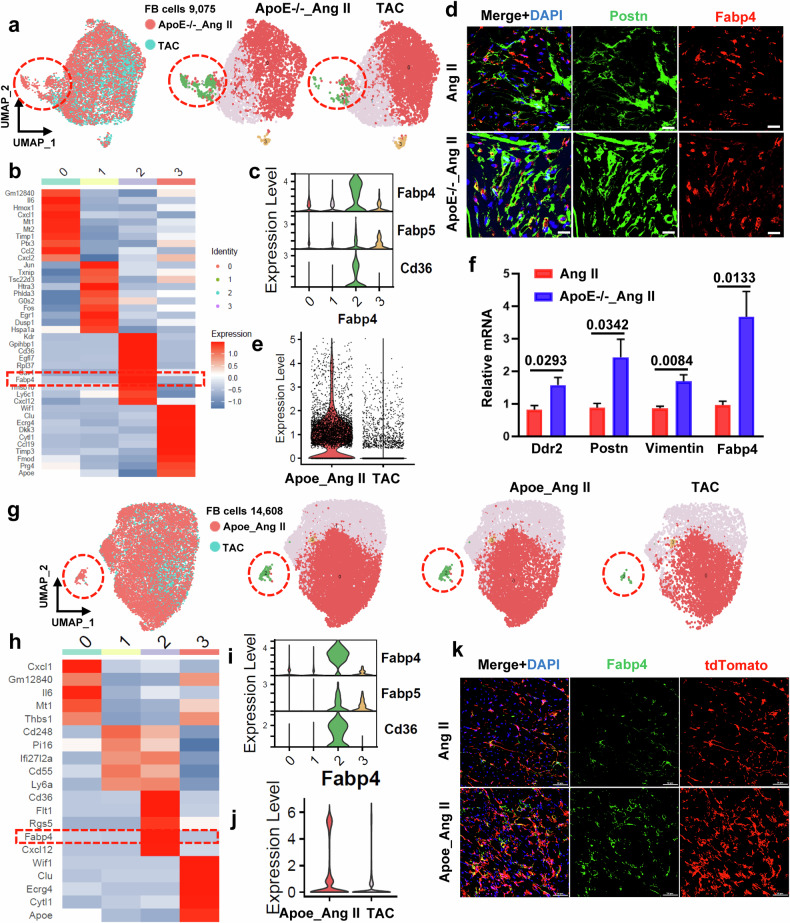
scRNA-seq analysis revealed the role of lipid metabolism disturbance in myocardial fibrosis. **a** UMAP plot displaying the distribution of fibroblast subpopulations among different datasets. **b** Heatmap showing the expression of the top ten differentially expressed genes in each cell subcluster. **c** Violin plot showing the expression of selected marker genes in each subcluster. **d** Representative images showing specific cell identification by staining for Postn and Fabp4. Scale bars = 20 μm. **e** Violin plot showing the expression of FABP4 in fibroblasts in the two groups. **f** mRNA levels of fibroblastic markers (Postn, Vimentin, DDR2 and Fabp4) in the hearts of mice. GAPDH was used as an internal control. Data are presented as the mean ± SEM, *n* = 4. *P* < 0.05 was considered to indicate statistical significance. **g** Heatmap showing the expression of the top ten differentially expressed genes in each cell subcluster from the tdTomato+ cell dataset of these two groups. **h**, **i** Violin plot showing the expression of selected marker genes in each subcluster. **j** Representative images showing specific cell identification by staining for tdTomato and Fabp4. Scale bars, 50 μm. **k** Representative images showing specific cell identification by staining for Fabp4 and tdTomato. Scale bars = 50 μm.

Previous data have shown that metabolic genes related to lipid metabolism are concentrated in fibroblast subsets, and we focused on fibroblasts to discover the differences between these two groups. The extracted fibroblast population was further divided into four subclusters (Fig. 5a). The UMAP plots displayed a significant dispersion of fibroblast clusters, highlighting a considerable degree of fibroblast heterogeneity (Fig. 5a, b). Notably, in our datasets, we found a specific subgroup (Cluster 3, termed FABP4^+^ fibroblasts) with high FABP4 expression among all subgroups of fibroblasts (Fig. 5a–c); compared to those in the TAC group, FABP4^+^ fibroblasts changed most significantly in the ApoE^-/-^_Angiotensin II group (Fig. 5a), and FABP4 expression significantly increased (Fig. 5e). Furthermore, compared to those in the Cd34-CreERT2; Rosa26-tdTomato (Angiotensin II 4 W) group, the expression of fibroblast markers (including Postn^+^, Fabp4^+^, and Fabp4^+^ Postn^+^) was increased in the heart tissues of Cd34-CreERT2; Rosa26-tdTomato&ApoE^-/-^ (Angiotensin II 4w) mice during disease progression, as shown by immunofluorescence staining (Fig. 5d, Supplementary Fig. 9a).

In addition, by examining the expression levels of fibroblast markers (including Postn, Vimentin, Ddr2 and Fabp4) in total fibroblasts isolated from the mouse heart, we found that the expression levels of fibroblast markers (such as Postn, Vimentin, Ddr2 and Fabp4) were greater in the ApoE^-/-^_AngII group than in the AngII group without the ApoE^-/-^ background (Fig. 5f). These results suggest that hyperlipidemia can accelerate fibroblast generation (especially FABP4^+^ fibroblasts) and accelerate myocardial fibrosis.

To further examine the effect of hyperlipidemia on CD34^+^ cells and CD34-lineage fibroblasts, we extracted fibroblast populations from the tdTomato^+^ cell single-cell dataset of these two groups and further clustered them into four subclusters. Similar to the previous results, we found a specific subgroup (Cluster 2, termed FABP4^+^ fibroblasts) with high FABP4 expression (Fig. 5g–i). Furthermore, compared to the TAC group, FABP4 expression in Cluster 2 was significantly increased (Fig. 5j). Additionally, compared to the Cd34-CreERT2; Rosa26-tdTomato (Angiotensin II 4W) group, the expression of fibroblast markers (Fabp4^+^ and Fabp4^+^ tdTomato^+^) was increased in the Cd34-CreERT2; Rosa26-tdTomato&ApoE^-/-^ group (Angiotensin II 4w), as shown by immunofluorescence staining (Fig. 5k, Supplementary Fig. 9a). These results suggest that hyperlipidemia can accelerate the differentiation of CD34^+^ cells into fibroblasts (especially FABP4^+^ fibroblasts).

### Non-bone marrow CD34^+^ cells differentiate into fibroblasts to promote cardiac fibrosis

**Fig. 6:**
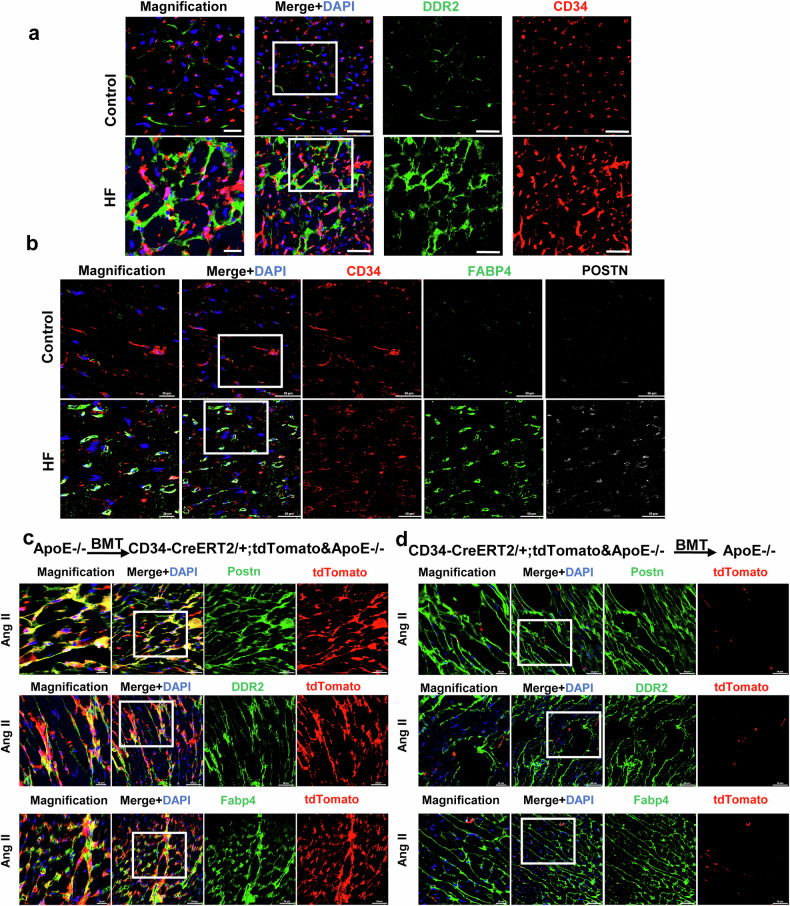
Non-bone marrow CD34+ cells differentiated into fibroblasts to promote fibrosis in cardiac hypertrophy. **a** Immunofluorescence staining of fibroblast markers (DDR2 and CD34) in the human heart. Scale bars, 50 μm and 20 μm in magnified images. **b** Representative images showing specific cell identification by staining for CD34, FABP4, and POSTN in the human heart. Scale bars, 50 μm and 20 μm in magnified images. **c** Immunofluorescence staining of tdTomato and fibroblast markers (Postn, DDR2, and Fabp4) in the bone marrow transplant of ApoE-/- to CD34-CreERT2;Rosa26-tdTomato&ApoE^-/-^ mice. *n* = 8 per group. Scale bars, 50 μm and 20 μm in magnified images. **d** Immunofluorescence staining of tdTomato and fibroblast markers (Postn, DDR2, and Fabp4) in bone marrow transplant of CD34-CreERT2; Rosa26-tdTomato &ApoE^-/-^ mice to ApoE^-/-^. *n* = 8 per group. Scale bars, 50 μm and 20 μm in magnified images. BMT bone marrow transplantation. HF heart failure.

Previous mouse data have demonstrated that CD34^+^ cells can differentiate into both fibroblasts and FABP4+ fibroblasts in mouse models. However, what occurs in the human heart is unclear. We stained heart sections from healthy people and patients with heart failure (with a history of hypertension and hyperlipidemia) using immunofluorescence and found that CD34 costaining for DDR2, POSTN, and FABP4 was greater in the heart failure group than in the control group (Fig. 6a, b).

To explore the origin of CD34-derived fibroblasts in cardiac hypertrophy, we established chimeric mice by transplanting bone marrow cells from wild-type ApoE^-/-^ mice into irradiated Cd34-CreERT2;Rosa26-tdTomato-ApoE^-/-^ mice. Subsequently, the chimeric mice were treated with tamoxifen and underwent surgery, and the cells were collected four weeks after the surgery for analysis (Supplementary Fig. 9a). Immunofluorescence staining was subsequently performed. As shown in Fig. 6c, in the bone marrow transplanted from ApoE^-/-^ to Cd34-CreERT2;Rosa26-tdTomato-ApoE^-/-^ mice, a significant number of fibroblast markers (Postn^+^, DDR2^+^, and FABP4^+^) were costained with tdTomato, proving that nonbone marrow CD34^+^ cells serve as the primary source for replenishing the fibroblast pool in cardiac fibrosis (Fig. 6c, Supplementary Fig. 9b–c). In contrast, in the bone marrow of ApoE^-/-^ mice transplanted from Cd34-CreERT2; Rosa26-tdTomato-ApoE^-/-^ to ApoE^-/-^ mice, the costaining of fibroblast markers (Postn^+^, DDR2^+^, and FABP4^+^) with tdTomato was rare (Fig. 6d, Supplementary Fig. 9b, c). By combining the fluorescence staining data from these two groups (Fig. 6c, d), the data strongly suggest that fibroblasts derived from non-bone marrow CD34+ cells play a significant role as major participants in cardiac fibrosis.

### Inducible ablation delineates the role of CD34^+^ cells in cardiac fibrosis

To further clarify the role of CD34+ cells in myocardial remodeling in response to angiotensin II, we employed a CD34+ cell depletion system to determine the involvement of CD34^+^ cells in fibrosis. To achieve the targeted elimination of CD34+ cells in vivo, we generated Cd34-CreERT2, Rosa26-eGFP-DTA, and ApoE^-/-^ (Cre/DTA group) mice and subsequently administered tamoxifen to induce recombination for DTA expression in CD34+ cells^12,28^.

**Fig. 7:**
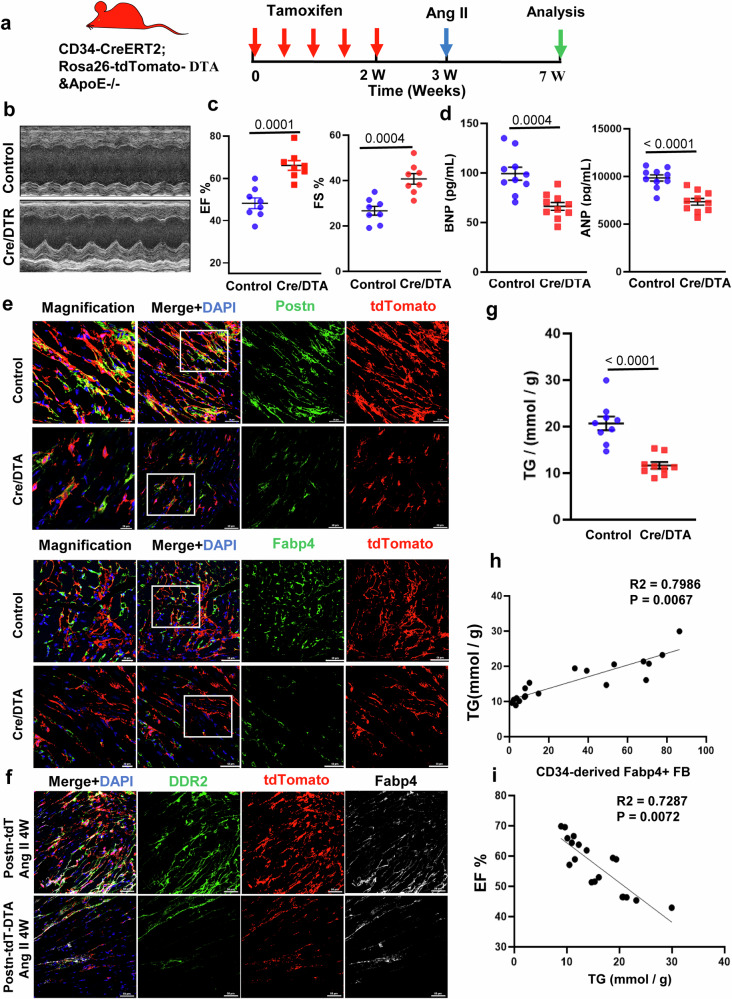
Inducible ablation delineates the role of cd34+ cells in cardiac fibrosis. **a** Schematic showing CD34-CreERT2;Rosa26-tdTomato/DTA&ApoE^-/-^ mice. The experimental scheme in which Cre/DTA mice were given tamoxifen for 1 week before sham or AngII surgery. Heart tissue was harvested 4 weeks after surgery. **b** Representative echocardiography of CD34-CreERT2;Rosa26-tdTomato (control) and CD34-CreERT2;Rosa26-tdTomato/DTA (Cre/DTA) mice at 4 weeks after surgery. **c** Echocardiographic measurements of the left ventricle ejection fraction (EF) and fractional shortening (FS) in the control group and Cre/DTA group 4 weeks after surgery. The data are presented as the mean ± SEM; n = 8 for the Cre/DTA group. *P* < 0.05 was considered to indicate statistical significance. **d** ELISA detection of serum ANP and BNP in the control group and Cre/DTA group at 4 weeks after surgery. Data are presented as the mean ± SEM, n = 10. *P* < 0.05 was considered to indicate statistical significance. **e** Representative immunostaining images showing tdTomato, Postn and Fabp4 staining in the control group and Cre/DTA group. Scale bars, 50 μm and 20 μm in magnified images. **f** Representative immunostaining images showing the tdTomato, DDR2, and Fabp4 staining in the two groups. Scale bars, 50 μm and 20 μm in magnified images. Total triglyceride (TG) levels in mouse heart tissue were measured. Data are presented as the mean ± SEM, *n* = 9. *P* < 0.05 was considered to indicate statistical significance. **g** Measurement of total triglycerides (TG) in mouse heart tissue, Data represent mean ± SEM, *n* = 9. *P* < 0.05 was considered to be statistically significant. **h** Correlation analysis of CD34-derived Fabp4+ fibroblasts and triglycerides (TG) levels. **i** Correlation analysis of triglycerides (TG) and the left ventricle ejection fraction (EF).

Surgical procedures were subsequently performed on Cre/DTA mice, both those treated with tamoxifen and those not treated with tamoxifen (Fig. 7a). Interestingly, the left ventricular EF and FS were significantly increased (Fig. 7b, c), while the levels of atrial natriuretic peptide (ANP) and B-type natriuretic peptide (BNP) in the serum were substantially reduced in the Cre/DTA group, suggesting a significant improvement in cardiac function (Fig. 7d). Additionally, Masson and Sirius red staining of cardiac sections revealed a reduction in fibrosis severity in the Cre/DTA group (Supplementary Fig. 10a, b). Furthermore, immunofluorescence staining revealed a significant decrease in the number of CD34-derived fibroblasts (including Vimentin, Postn, and FABP4) in the Cre/DTA group compared to the control group (Fig. 7e, Supplementary Figs. 10c, 11a). Furthermore, the percentage of CD34-lineage fibroblasts (PDGFRa+tdTomato+) in heart tissues was also greater in the Cre/DTA group than in the control group according to flow cytometry analysis (Supplementary Fig. 10d).

To further demonstrate the effect of fibroblast reduction on cardiac function, Postn-CreERT2;Rosa26-tdTomato and Postn-CreERT2;Rosa26-tdTomato-DTA mice were generated (Supplementary Fig. 11b, c). We then examined the effect of genetic ablation of cardiac fibroblasts following angiotensin II treatment on cardiac function. Similar to the results of previous studies^29,30^, as shown in Supplementary Fig. 11d, genetic ablation of Postn^+^-activated fibroblasts significantly improved cardiac function (Supplementary Fig. 11d). In addition, upon immunofluorescence staining, the expression of FABP4 and DDR2 was also significantly reduced after genetic ablation of Postn-activated fibroblasts (Postn-tdT-DTA-Angiotensin II group) when compared to the Postn-tdT-Angiotensin II group (Fig. 7f). These findings indicate that CD34+ cells play a role in myocardial fibrosis by differentiating into fibroblasts and that the deletion of CD34+ cells can alleviate the cardiac fibrotic process and lead to an improvement in cardiac function.

Recent research has suggested that the excessive accumulation of TG may lead to cellular lipotoxicity, which could contribute to the progression of renal fibrosis^31–33^. In contrast, our results show that lipid metabolism disorders may play a role in fibrosis. Therefore, we measured the lipid content (TG) in heart tissue. Surprisingly, as shown in Fig. 7g and Supplementary Fig. 12a, b, the TG content of the heart tissue and the serum in the Cre/DTA group was significantly lower than that in the control group (Fig. 7g, Supplementary Fig. 12a, b), suggesting that the deletion of CD34^+^ cells can reduce the amount of TG in the heart and further reduce lipid toxicity in the heart.

Studies have shown that FABP4, a member of the fatty acid-binding protein family, plays a pivotal role in lipid metabolism, facilitating processes such as lipid storage, distribution, transport, decomposition, and metabolism. Furthermore, FABP4 has been associated with lipid metabolism disorders^3,34^. Therefore, the decrease in TG content in the Cre/DTA group may be related to a significant decrease in CD34^+^ cell-derived FABP4^+^ fibroblasts. To further clarify the relationships among CD34^+^ cell reduction, myocardial fibrosis, TG content, and cardiac function, we conducted a series of correlation analyses and found that CD34-derived fibroblasts were significantly correlated with cardiac function (Supplementary Fig. 12c, d).

More importantly, CD34-derived FABP4 was significantly positively correlated with the TG content of heart tissue, while the TG content of heart tissue was significantly negatively correlated with cardiac function (Fig. 7h, i). From the above data, we can conclude that CD34^+^ cells can accelerate the accumulation of lipids in the heart through differentiation into FABP4-producing cells, resulting in lipid toxicity, which leads to the progression of heart failure.

### Heart-derived CD34^+^ cells differentiate into fibroblasts in the lipid microenvironment and pressure overload model

**Fig. 8:**
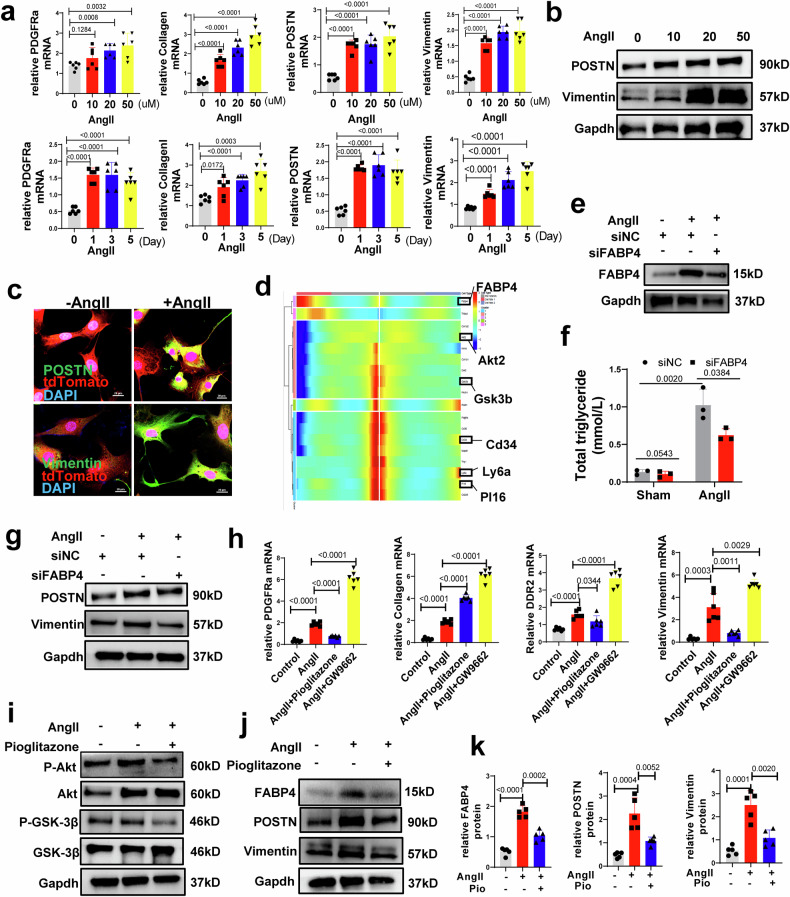
Heart tissue-derived CD34+ cells from ApoE^-/-^ mice differentiated into fibroblasts. **a**, **b** Heart-derived CD34+ cells of ApoE^-/-^ mice treated with different concentrations of AngII (0 μM, 10 μM, 20 μM, and 50 μM) at different times (day 0, day 1, day 3, and day 5). **a** mRNA levels of fibroblastic markers; GAPDH was used as an internal control. Data represent mean ± SEM, n = 6. P < 0.05 was considered to be statistically significant. **b** Protein expression of fibroblastic markers after treatment with AngII, data represent mean ± SEM, n = 4. P < 0.05 was considered to be statistically significant. **c** Immunofluorescence staining of POSTN and Vimentin in tdTomato positive cells after 3 days of AngII stimulation (50 μM) (Scale bar= 50 μm). **d** Heatmap of the significantly changed genes (*P* < 0.01) discovered by the BEAM function from monocle in the branch point 3 in Fig. 4a. Cd34, ly6a, and Pi16 genes were detected in Gene Module 1, and the FABP4 gene in module 3. **e**–**g** CD34+ cells were transfected with FABP4 siRNA and then treated with AngII (50 μM) for 3 days. Data represent mean ± SEM. **e** Protein expression of FABP4 was determined by western blotting; **f** Total triglyceride in the cells, *n* = 3. **g** Protein expression of fibroblastic markers was determined by western blotting; **h**–**k** CD34+ cells were treated with PPARγ agonist pioglitazone (10 nM) and PPARγ antagonist GW9662 and then treated with AngII (50 μM) for 3 days: **h** mRNA levels of fibroblastic markers and ECM proteins; GAPDH was used as an internal control, *n* = 6; *P* < 0.05 was considered to be statistically significant. **i** Protein expression of Akt and GSK3beta was determined by western blotting. **j** Protein expression of FABP4 and fibroblastic markers were determined by western blotting. **k** Quantitative data of FABP4 and fibroblastic markers is shown; GAPDH was used as an internal control, *n* = 5. *P* < 0.05 was considered to be statistically significant.

Our data suggest the possible involvement of resident CD34^+^ cells in fibroblast generation in the presence of coexisting hypertension and lipid metabolic syndrome. We hypothesized that cardiac-resident CD34+ cells might possess an increased potential to differentiate into fibroblastic cells. To investigate this possibility, we isolated heart-resident CD34^+^ ApoE^-/-^ cells from Cd34-CreERT2;Rosa26-tdTomato ApoE^-/-^ mice (see Methods) and confirmed their identity through CD34 immunofluorescence staining (Supplementary Fig. 13a). Subsequently, CD34^+^ ApoE^-/-^ cells were exposed to angiotensin II, utilizing a concentration and time gradient for further analysis. In our investigation, we observed significant upregulation of various fibroblastic hallmark markers, including PDGFRα, collagen I, Vimentin, and periostin (Postn), in CD34^+^ ApoE^-/-^ cells treated with angiotensin II at a concentration of 20 µM over a 3-day period (Fig. 8a). Fibroblast markers, such as Postn and Vimentin, increased after angiotensin II stimulation as the concentration increased and exhibited a significant increase at a concentration of 50 µM for 3 days (Fig. 8b, c, Supplementary Fig. 13b, c). These findings suggest that heart-resident CD34+ ApoE^-/-^ cells can be induced to differentiate into fibroblastic cells by angiotensin II in vitro. To better understand the underlying mechanism, we analyzed the scRNA-seq data, which indicated that FABP4 may play a crucial role in this process (Fig. 8d). Notably, recent studies have demonstrated the significant involvement of FABP4 in metabolically driven chronic inflammation in cardiovascular disease^4^. To clarify the potential role of FABP4 in regulating lipid metabolism in fibroblastic cells derived from CD34^+^ ApoE^-/-^ cells, FABP4 siRNA (Fig. 8e, Supplementary Fig. 13d, e) or a vector (Supplementary Fig. 13f, g) for FABP4 was transfected into CD34^+^ cells to downregulate or upregulate FABP4 expression, and the level of total TG in the cells was measured. Total TG increased in CD34^+^ ApoE^-/-^ cells treated with angiotensin II, while FABP4 siRNA blocked the storage of TG in the cells (Fig. 8f). Our results also showed that FABP4 inhibition decreased the expression of fibroblastic markers, such as collagen I and periostin *(Postn)*, in CD34^+^ ApoE^-/-^ cells treated with angiotensin II (Fig. 8g). FABP4 overexpression increased the levels of TG and fibroblast markers in the cells (Supplementary Fig. 13h–k).

FABP4 influences PPARγ expression and nuclear translocation^35^, and the PPARγ pathway is involved in fibrosis in multiple diseases^36–38^. In addition, as mentioned previously, FABP4^+^ fibroblasts (Cluster 6), a subgroup of fibroblasts in the tdTomato^+^ cell population (Fig. 4e), were enriched in the PPAR signaling pathway (Fig. 4f). Therefore, we interfered with the PPARγ pathway in CD34^+^ ApoE^-/-^ cells before angiotensin II treatment. The PPARγ antagonist GW9662 increased the expression of *PDGFRα*, *collagen I*, *Vimentin*, *Postn, and DDR2* in CD34^+^ cells treated with angiotensin II, while the PPARγ agonist pioglitazone blocked the expression of *PDGFRα*, *collagen I*, *Vimentin*, *Postn*, and *DDR2* (Fig. 8h, j, k). CD34^+^ ApoE^-/-^ cells subjected to the PPARγ agonist pioglitazone expressed lower levels of Akt and GSk-3beta under angiotensin II stimulation (Fig. 8i). Interestingly, the increase in FABP4 expression in cells subjected to angiotensin II stimulation was blocked after pioglitazone pretreatment (Fig. 8j–k). To further clarify this result, the effects of antagonists and agonists were verified in mouse models. Briefly, the PPAR-gamma antagonist GW9662 and agonist pioglitazone were administered to CD34-CreERT2&ApoE^-/-^ mice with angiotensin-induced hypertension (Supplementary Fig. 14a–e). Hypertension promoted cardiac fibrosis and induced cardiac dysfunction. Pioglitazone alleviated the increase in fibrosis markers, which is consistent with the in vitro data (Supplementary Fig. 14a–e).

## Discussion

It is well known that tissue fibroblasts exert their effect on cardiac fibrosis during heart failure, but the detailed mechanisms underlying the effects of altered microenvironment factors (lipid disorders and mechanical stress) remain unclear. Previous studies have shown that adverse left ventricular remodeling and myocardial fibrosis begin in parallel after blood pressure increases in animal models of systemic hypertension (refs.^39–42^). The mechanism of myocardial fibrosis in chronic systemic hypertension may involve different factors. For example, the initial response to mechanical stress due to hypertension-induced ventricular pressure overload results in quiescent fibroblast activation sensed through surface receptor integrins, leading to extracellular matrix protein accumulation. In addition to mechanical stress, this can be produced during hypertension, which can contribute to the process of myocardial fibrosis^43,44^. Nevertheless, the synergistic mechanism between hypertrophy and hyperlipidemia in fibrosis has not been fully elucidated.

Our previous study reported that non-bone marrow CD34+ cells are crucial for endothelial repair of injured arteries^24^. It is less known whether CD34^+^ cells play a role in endothelial regeneration/angiogenesis during cardiac remodeling. Several clinical studies have shown the potential benefits of the transplantation of CD34+ cells for treating myocardial ischemia or other heart diseases^45–49^, which is different from our study. We focused on the role of resident CD34+ cells in ventricular remodeling and the role of CD34+ cells in fibrosis. The clinical application of CD34+ cells in cardiovascular therapy is still controversial. In the present study, we provided evidence that CD34^+^ cells can differentiate into a special type of fibroblast called FABP4^+^ fibroblasts in the presence of hyperlipidemia and hypertension. Moreover, a disturbance in lipid metabolism can accelerate the formation of FABP4^+^ fibroblasts and accelerate myocardial fibrosis. Moreover, in a mouse model of heart failure, the depletion of CD34+ cells resulted in a reduction in FABP4+ fibroblasts, triglyceride (TG) content, and myocardial fibrosis and an improvement in cardiac function. Intriguingly, our findings indicate that CD34+ cells isolated from the heart undergo differentiation into fibroblasts through the PPARγ/Akt/Gsk3β pathway. These findings provide evidence that lipid metabolism disorders accelerate the differentiation of CD34^+^ cells into fibroblasts and induce myocardial fibrosis. In contrast, CD34^+^ cells can accelerate the accumulation of lipids in the heart by differentiating into FABP4^+^ fibroblasts, resulting in lipid toxicity that leads to the progression of heart failure.

The heart’s energy demands are met through the utilization of various substrates, such as carbohydrates, lipids, amino acids, and ketone bodies. The heart’s substrate preference changes during different stages of its life cycle and under various physiological and pathological conditions, allowing the heart to adapt to environmental changes^20,50^. During development, the heart relies heavily on aerobic glycolysis and lactate oxidation, with fatty acid oxidation playing a minor role. In contrast, the adult heart predominantly employs oxidative metabolism for ATP production, and fatty acids become the primary fuel source. However, in hearts experiencing pathological hypertrophy, they revert to a fetal metabolic profile, exhibiting increased reliance on glucose and reduced oxidative capacity. This metabolic shift leads to decreased oxidative metabolism, heightened oxidative stress, insulin resistance, lipid accumulation, and energy deprivation, collectively contributing to heart failure^51^. Consistent with this, our serum metabolomics data showed that lipid-related substances accounted for the majority of metabolic substances and that TG comprised the majority of the lipids; we also found a significant increase in TG in the heart failure group. These data support the concept that changes in lipid metabolism might initiate cardiac fibrosis.

Previous studies on metabolic changes in pathologically hypertrophic hearts have focused mainly on the effects of metabolic changes in cardiomyocytes (including a reduced energy supply to cardiomyocytes due to reduced fatty acid oxidation, oxidative stress, and disturbances caused by lipid accumulation) while ignoring the effects of metabolic changes on noncardiomyocytes (fibroblasts, stem cells, etc.)^20,52^. Most importantly, increased intracellular lipid accumulation plays a critical role in the onset of renal fibrosis by inducing lipotoxicity^53,54^. These results further suggest that the regulation of lipid metabolism may be an effective way to regulate fibrosis. An increasing body of evidence indicates that noncardiomyocyte cells play a crucial role in myocardial remodeling^55–58^. Cardiac fibrosis is recognized as one of the major contributors to myocardial remodeling^59,60^, and it has been closely associated with the progression of heart failure^61^. Therefore, we believe that lipid metabolism likely plays a significant role in the interplay between fibroblasts and myocardial fibrosis.

Based on our human scRNA-seq dataset and consistent with previous reports^12^, our findings revealed a noteworthy increase in the proportion of fibroblasts in the heart failure group compared to that in the control group. In addition, we found that active fibroblast markers (POSTN, THBS4, CILP, and FN1) were significantly elevated in the heart failure group. In our mouse scRNA-seq dataset, we observed a considerable increase in fibroblast markers (Vimentin, Ddr2, Postn) in the ApoE^-/-^ group compared to the control group. Fibroblast markers in the ApoE^-/-^ Angiotensin II group were significantly greater than those in the ApoE^-/-^ group. Together, these data suggest that hypertension combined with hyperlipidemia can further accelerate myocardial fibrosis compared to hyperlipidemia alone. Furthermore, we found that the expression of a new specific subgroup (Fabp4+ fibroblasts), which is associated with lipid metabolism and triglyceride metabolism, increased in patients with heart failure caused by the combination of coronary artery disease and hypertension. In addition, the number of FABP4+ fibroblasts changed most significantly in the HHD_HF group compared with the CHD_HF group. Consistent with the findings in humans, the change in FABP4^+^ fibroblasts was most significant in the context of hypertension combined with hyperlipidemia in mice. Surprisingly, the ApoE^-/-^_Angiotensin II group exhibited a significant increase in the expression of both FABP4 and fibroblast markers compared to the TAC group. These data suggest that the disruption of lipid metabolism (hyperlipidemia) can accelerate the production of FABP4+ fibroblasts, which are significantly increased when there is coexisting hypertension compared to hypertension or hyperlipidemia alone, thus accelerating myocardial fibrosis.

To illustrate the potential origin of this unique new fibroblast subgroup, we combined scRNA-seq and genetic lineage tracing and found that CD34 cells account for the production of Fabp4+ fibroblasts and are related to the regulation of lipid metabolism processes. Additionally, we found that the disturbance of lipid metabolism can accelerate the differentiation of CD34 cells into fibroblasts (especially FABP4^+^ fibroblasts), which is significantly increased when lipid metabolism disruption is combined with hypertension in comparison to hypertension or lipid metabolism disruption alone.

Subsequently, we conducted a thorough analysis of CD34+ cells from the human heart, revealing their ability to differentiate into fibroblasts and FABP4+ cells during myocardial remodeling. Consequently, these findings imply that CD34+ cells may be involved in fibrosis through their differentiation into FABP4+ fibroblasts, which distinguishes them from other cardiac fibroblasts involved in cardiac remodeling in lipid metabolism disorders (hyperlipidemia). This finding underscores the diverse functions and heterogeneous nature of cardiac fibroblasts in the fibrotic process. To determine the source of CD34-derived fibroblasts in a model of hypertension combined with hyperlipidemia, bone marrow transplantation was performed, and the results indicated that non-bone marrow CD34+ cells can differentiate into Fabp4+ fibroblasts and may be the major participants in cardiac fibrosis. Moreover, hyperlipidemia can accelerate this differentiation process. This result further corroborated our previously published evidence that non-bone marrow CD34+ cells, rather than bone marrow CD34+ cells, play a vital role in differentiating into fibroblasts in TAC surgery-induced heart failure^12^.

Importantly, to further clarify the differentiation process of CD34+ cells into fibroblasts rather than the proliferation of CD34^+^ cells in myocardial fibrosis, we constructed CD34Dre, PostnCreERT2, and R26-RSR-LSL-TdT dual-recombinase-activated lineage tracing mice^62^. The advantage of this system is that it eliminates ectopic expression by excluding the labeled progeny cells in the Cre-LoxP recombinant system that are not of the targeted cell origin (if there is a trace of Cre expression in nontargeted cells). Therefore, the recombinant system (Dre-rox) can increase the accuracy of Cre-LoxP-mediated lineage tracing results and provide more accurate lineage tracing. We found that fibroblasts were mainly derived from the differentiation of CD34^+^ cells rather than from their own proliferation during myocardial fibrosis.

As previously mentioned, a novel discovery in mouse models revealed the potential of CD34+ cells to differentiate into FABP4+ fibroblasts, contributing to cardiac fibrosis. It is crucial to validate these findings and further elucidate the mechanisms underlying cell differentiation and activation. In our in vitro experiments, we observed that CD34+ cells from healthy hearts could differentiate into fibroblasts expressing various fibroblastic markers and producing collagen when exposed to angiotensin II stimulation. PPARγ plays a vital role in regulating fatty acid transport, oxidation, and decomposition by controlling the expression levels of fatty acid transporters and fatty acid binding proteins^63^. Furthermore, FABP4^+^ fibroblasts of the CD34 lineage were related to the PPARγ signaling pathway according to GO enrichment analysis; moreover, pseudotime analysis of CD34^+^ lineage cells and in vitro experiments revealed the crucial role of the PPARγ/Akt/Gsk3β pathway in regulating the differentiation of CD34+ cells into FABP4^+^ fibroblasts.

Recently, studies have demonstrated that genetic ablation of cardiac fibroblasts following hypertensive or ischemic injury leads to a reduction in fibrosis and an improvement in heart function in mice^29,30,64^. However, in clinical practice, there is currently no therapeutic technique available to directly target excessive fibrosis, and only a limited number of interventions have shown efficacy in enhancing cardiac function and clinical outcomes in heart failure patients with impaired cardiac compliance. To elucidate the role of CD34+ cells in myocardial remodeling under conditions of pressure overload combined with hyperlipidemia, surgical procedures were performed on Cre/DTA model animals. To our surprise, the data demonstrated that when CD34+ cells were partially depleted, there was a notable decrease in the fibrotic area and a significant improvement in cardiac function compared to those of the control animals.

Recent studies have demonstrated that FABP4 inhibitors or FABP4 deficiency can ameliorate lipid deposition and regulate dyslipidemia and lipotoxicity^65–67^. Thus, partial elimination of CD34+ cells leads to a significant reduction in FABP4+ fibroblasts within the heart, resulting in decreased TG content, reduced myocardial fibrosis, and improved cardiac function. Taken together, CD34^+^ cells can accelerate the accumulation of lipids in the heart by differentiating into FABP4^+^ fibroblasts, resulting in lipid toxicity, which leads to the progression of heart failure. These findings strongly support the possibility of directly targeting CD34+ cells in the treatment of myocardial fibrosis in the presence of hyperlipidemia.

To summarize, our study utilized scRNA-seq of hearts from mice and humans, in addition to lineage tracing techniques, to elucidate the crucial involvement of CD34+ cells in myocardial remodeling during hyperlipidemia. We systematically characterized the cellular landscape of myocardial fibrosis at single-cell resolution and generated a comprehensive cell atlas encompassing CD34+ cells and fibroblasts. We identified a fibroblast subset (FABP4^+^ fibroblasts) associated with lipid metabolism and demonstrated that lipid metabolism disorders can accelerate fibroblast formation and fibrosis. Moreover, the pivotal role of the PPARγ/Akt/Gsk3β pathway in differentiating CD34+ cells into FABP4+ fibroblasts was established. Overall, our study not only provides a valuable reference regarding the cell types implicated in heart failure but also reveals fresh perspectives on the pathogenesis of cardiac fibrosis, offering potential avenues for developing future therapeutic approaches for heart failure.

## Supplementary information

Supplementary Information

## Data Availability

All of the data needed to evaluate the conclusions in the paper are presented in the paper and/or the Supplementary Materials. The scRNA-seq data of the mouse and human hearts are available for reproducing the results and have been uploaded. The data that support the findings of this study are available from the Gene Expression Omnibus (GEO) (GSE243389 and GSE247468).
