# Supplementary material for: Altered lipid metabolism promoting cardiac fibrosis is mediated by CD34+ cell-derived FABP4+ fibroblasts
Source: Exp Mol Med. 2024 Aug 29;56(8):1869–86. doi: 10.1038/s12276-024-01309-9 (PMC11372182; doi:10.1038/s12276-024-01309-9)
Supplement: Supplementary file 1 — Supplementary Information [file 12276_2024_1309_MOESM1_ESM.pdf]

Supplementary Information

This Supplementary Information contains:

Supplementary materials and methods ……………………………...……………2

Supplementary figures

Supplementary Fig.1 to Fig.14 …………………...………………………………10

Supplementary materials and methods

Angiotensin II (Ang II) induced cardiac hypertrophy Model

Animals were fed a standard laboratory diet with free access to food and water and kept in a temperature (22 ± 1°C) and humidity (65–70%) controlled room, with a 12-h light–dark cycle. All mice generated or purchased were housed in Laboratory Animal Center of Zhejiang University (Hangzhou, China) for at least one week before use. Tamoxifen (Sigma C8267) dissolved in corn oil (20 mg/mL) was administered via gavage at indicated time points (at 0.1-0.15 mg/gram of mouse body weight) over a period of two weeks to activate tdTomato labeling.

Adult male mice weighing 25±5g were randomly allocated to different groups. Animals were anesthetized by Isoflurane and Adequacy of anesthesia was monitored by using the corneal and withdrawal reflexes. Then endotracheal intubation was performed and the mouse was connected to a ventilator. Ventilation frequency was kept at 125-150 breaths per minute with a tidal volume between 135 and 150 μL. The chest was entered in the second intercostal space at the top left aortic arch, then the transverse aorta was isolated, and aortic constriction was performed by tying a 7-0 nylon suture ligature against a 27-gauge blunt needle. The needle was then removed to yield a constriction 0.4 mm in diameter. In sham-operated control mice, the entire procedure was identical except that aortic constriction was not performed. The chest tube was used to evacuate the pneumothorax, and it was removed once negative pressure was reestablished. The chest was closed in layers using 5-0 sutures. Body temperature was maintained at 37.0 °C throughout the whole surgical procedure using a self-regulating heating pad. For analysis, animals were randomly divided to sham and 4 weeks post-operation groups. After Ang II surgery, mice were only excluded/ euthanized humanely in time when the animal health condition was poor.

Echocardiography

In vivo cardiac morphology was assessed by transthoracic ultrasonography imaging with Vevo 770 high-resolution ECHO system equipped with a 35 MHz transducer. Mice were sedated with isoflurane vaporized (2.5% for induction, 1.0% for maintenance) in oxygen and adjusted the inhalational flow of isoflurane to maintain their heart rate at 450–550 beats/min. Left ventricular function was assessed by M-mode scanning of the left ventricular chamber, and left ventricular size (end-diastolic and end-systolic dimension), wall thickness (posterior wall thickness) were measured for at least three beats from each projection and averaged. All mice underwent echocardiography 0, 4 weeks after surgery. Studies and analysis were performed blinded to heart condition.

Histology and Trichrome Staining

Hearts were harvested and embedded in paraffin, then cut into 5 μm thick

sections. Haematoxylin and eosin (H&E), Masson's trichrome staining and Picro-Sirius red staining were performed according to manufacturer's instructions.

Immunofluorescence Staining

Heart tissues were fixed for 6-8 hours at 4°C in 4% paraformaldehyde in PBS, washed in PBS, immersed in a solution of 30% sucrose in PBS at 4°C overnight, embedded in Tissue-Tek OCT Compound. Frozen tissue slides were prepared at 5-μm thickness using a cryostat (Leica) and stored at -80°C. slides were dried at room temperature for 20 minutes, washed 3 times for 10 minutes each in PBS, permeabilized in PBS containing 0.25% Triton X-100 (Fisher Scientific) for 10 minutes at room temperature followed by washing in PBS for 5 minutes. The tissue slides were incubated with blocking buffer (5% BSA in PBS) for 60 minutes at room temperature. The tissue slides were then incubated with primary antibodies diluted in blocking buffer overnight at 4 °C followed by 30 min at room temperature. After washing three times for 10 minutes with PBS, the tissue slides were incubated with the secondary antibody for 60 minutes at room temperature, washed three times for 10 minutes with PBS, and then mounted with DAPI-containing mounting medium. And all slides were mounted with anti-fade mounting medium. The immunostained slides were observed and analyzed using Leica confocal microscope. As for quantification of immunostaining images, we used the Image J to analysis the images from 3-6 different sections. Primary antibodies used in this study were as follows: Vimentin (abcam, ab8978), DDR2 (R&D, MAB25381), PDGFRA (R&D, AF1062), POSTN (R&D, AF2955), THBS4 (R&D, AF2390), CD34 (abcam, ab81289), CD31 (R&D, AF3628), RFP antibody (Rockland, 600-401-379). Alexa Fluor-conjugated secondary antibodies used in this study included Donkey anti-Mouse IgG (Invitrogen, A21202 for Alexa Fluor 488), Donkey anti-Goat IgG (Invitrogen,A11055 for Alexa Fluor 488, A21447 for Alexa Fluor 647), Donkey anti-Rabbit IgG (Invitrogen, A31572 for Alexa Fluor 555).

Bone Marrow Transplantation (BMT)

Bone marrow transplantation was performed as previously described. Mouse hind limbs were collected and bisected by cutting through the knee joint. Muscle and connective tissues of both the femur and tibia were removed by scraping the diaphysis of the bone. After cleaning, the femur and tibia were cut just at the end of the marrow cavity and the marrow was sluiced out by RPMI 1640 (Invitrogen) followed by filtering through 40 μm cell strainers. Cells were resuspended in serum-free RPMI 1640 before transplantation. A lethal dose of whole-body irradiation (9.0-9.5 Gy) was administered to recipient mice. Six hours later, irradiated recipient mice received 5×10^6 donor bone marrow cells via tail vein injection to form chimeric mice.

Isolating fibroblasts from the heart in mouse and human

The cell isolation methods were described previously. Briefly, the heart segments were washed tress times with PBS supplemented with 1% penicillin/streptomycin(P/S). Carefully pump remaining blood out of the hearts. Cut vessels and remaining connective tissue away from the ventricle. Cut each heart into small pieces (1-2mm^3). For enzymatic digestion methods, multi tissue dissociation kit2 (#130-110-203) was used for adult heart dissociation and volumes given below for up to 5 adult mouse hearts (maximal 500mg) per C tube and 2.5ml of enzyme mix. Transfer harvested tissue into the gentleMACS C tube and add 2.5ml of enzyme mix. Invert C tube and place it with the cap down. Run program Multi_G through the gentleMACS Octo Dissociator with Heaters. After termination of the program, detach C Tube from the gentleMACS Dissociator and add 7.5ml of cell cultere medium with 20% FBS. Resuspend asmple and apply the cell suspension to a MACS smartStrainer (70um) with 3ml of cell culture medium with 20% FBS. Proceed with the red blood cell lysis and cells were then seeded onto cellstart-coated dishes and maintained at 37℃ in an incubator with 5% CO_2. All steps for cell culture experiments subsequent to tissue dissociation were performed under sterile conditions.

Single-cell Dissociation and Cell Sorting

For isolation of single cells from the mouse or human heart, a similar protocol was followed as previously described. After collecting the heart of sham or Ang II-operated mice or human hearts, the tissue was minced into fine pieces using a scalpel and transferred into a glass vial with 1.5 ml of cold digestion buffer (Liberase, Roche). The tissues were digested by either using a shaking (100 rpm) 37°C waterbath for 15 min. The obtained cell suspension was gently pipetted up and down (10 x) and transferred onto a 70μm cell strainer placed on top of a 50ml Falcon tube. The tissue was gently rubbed through the strainer using the plunger of a 1ml syringe, after which the strainer was rinsed with 8.5ml of DMEM to obtain a total volume of 10 ml, which was centrifuged for 6 min at 4°C at 300 xg. After the supernatant was removed, non-cardiomyocytes samples pelleted cells were suspended in red blood cell lysis buffer (Miltenyi Biotec) to lyse red blood cells. After washing with PBS, cells were filtered with 40 μm filter and then centrifuged at 300xg for 5 minutes. Cells resuspended in PBS were stained with LIVE/DEAD Fixable Near-IR (APC/Cy7 channel) Dead Cell Stain Kit (Invitrogen; L34975, 1:1000) and Hoechst 33342 (Invitrogen; H3570, 1:1000) for 20 minutes. Unstained cells and cells staining with only 1 fluorochrome prepared concomitantly served as control. After 1 wash in PBS for 5 minutes, cells were resuspended in PBS and then sorted with BD FACSARIA II. Nucleated live cells (Hoechst+/APC/Cy7− population) were sorted into PBS with 0.04% BSA for subsequent scRNA-seq. For isolation of single tdTomato+ cells from the heart, a similar protocol was used as describe above.

FACS Analysis

To isolate cells from freshly collected cardiac tissue, the heart was first cut into pieces. Subsequently, the tissue was treated with a digestion buffer (Liberase, Roche) at 37°C for 15 minutes. The resulting primary cells were then passed through a 40-μm cell strainer to obtain a single-cell suspension. To remove red blood cells, the cell samples were treated with red blood cell lysis buffer (Miltenyi Biotec). Following this, all prepared cell samples were suspended in PBS and incubated with conjugated antibodies for 30 minutes, as indicated. The antibodies used in this study included LIVE/DEAD Fixable Near-IR Dead Cell Stain Kit (Invitrogen, L34975), Hoechst 33342 (Invitrogen, H3570), PDGFRA-APC (Invitrogen, 17-1401), and CD34-FITC (BD Biosciences, 560238). Cell analysis was performed using a BD FACSVerse Flow Cytometer (BD Biosciences), and the resulting data was analyzed using FlowJo software.

Processing of scRNA-seq Data

Raw scRNA-seq data was processed using the 10x Genomics Cell Ranger software (version 3.1.0). The BCL files obtained from the Illumina NextSeq platform were processed to Fastq files using the Cell Ranger mkfastq program. Analyses of processed scRNA-seq data were carried out in R version 3.6 or 4.0 using the Seurat suite versions3.2.3. R scripts containing the steps used for processing and clustering the data for each individual data-set. For all data-sets, initial quality control filtering metrics were applied as follows: Low quality cells (<400 genes/ cell, >20000 genes/ cell and >10 % mitochondrial transcript presence/ cell) were excluded from downstream analyses.To remove the batch effect, the datasets collected from different samples were integrated using Seurat v3 with default parameters.

Data were then log-normalized for subsequent analysis. Dimensionality reduction using PCA was undertaken to explore transcriptional heterogeneity and perform cell clustering. 30 PCs were selected that explained more variability than expected by chance using heuristics detailed in vignettes associated with the Seurat software. PC loadings were used as input for a graph-based approach to cluster cells by cell type, and as input for UMAP for reduction to two dimensions for visualization purposes. Visualization of gene expression with violin plot, feature plot, dot plot, and heatmap was generated with Seurat function VlnPlot, FeaturePlot, DotPlot, DoHeatmap and ggplot2. Markers for a specific cluster against all remaining cells were found with function FindAllMarkers (Arguments: only.pos=TRUE, min.pct=0.25, logfc.threshold = 0.25).

Gene Ontology Analysis

Gene ontology (GO) and Kyoto encyclopedia of genes and genomes pathway analysis were performed with marker genes of each cluster found by FindAllMarkers function or enriched genes found by FindMarkers function with

average log (fold change) >0.25 on Metascape website (the database for annotation, visualization and integrated discovery).

Pseudotime Trajectory Analysis

For pseudotime analysis, we utilized Monocle (version 2.16.0). In summary, we reduced the dimensionality of our datasets using the "DDRTree" method and then ordered the cells along the pseudotime trajectory by utilizing the differentially expressed genes obtained from the Seurat analysis.

Human blood serum sample collection

We recruited eligible participants at the First Affiliated Hospital, Zhejiang University Medical School. Exclusion criteria included patients with severe liver and kidney disease, hematological disorders, psychiatric disorders, diabetes, autoimmune diseases, malignancies, thoracalgia due to aortic dissection or pulmonary embolism, and acute pericarditis. On the basis of clinical characteristics, electrocardiogram examinations, cardiac troponin T (cTnT) levels, Lipid related testing, blood pressure testing and coronary angiography. The serum samples (about 1 mL) were collected and stored at –80 ℃ immediately after collection. The Research Ethics Committee of the First Affiliated Hospital, Zhejiang University Medical School approved this study (Approval Reference No. 2021/330). The informed consent of all participants was obtained before they were enrolled in the study.

Nontargeted GC-MS-based Metabolic Profiling

The derivative samples underwent analysis using an Agilent 7890B gas chromatography system paired with an Agilent 5977A MSD system, both products of Agilent Technologies, Inc., based in Santa Clara, CA, USA. The separation of the derivatives was facilitated by a DB-5MS fused-silica capillary column (30 mm × 0.25 mm × 0.25 mm) from Agilent J & W Scientific in Folsom, CA, USA. Helium gas (purity >99.999%) was used as the carrier gas at a flow rate of 1 mL/min through the column. The injector temperature was set at 260 ℃ and the injection volume was maintained at 1L.

The temperature protocol for the oven started at 60 ℃, held for 0.5 minutes, followed by an increase to 125 ℃ at 8 ℃/min, then to 210 ℃ at 5 ℃/min, 270 ℃ at 10 ℃/min, and finally to 305 ℃ at 20 ℃/min, where it was held for 5 minutes. The MS quadrupole and the ion source temperatures were set at 150 ℃ and 230 ℃, respectively. The ionization method used was electron impact. Analytical data were collected in full-scan mode, covering a mass-to-charge ratio (m/z) range from 50 to 500, with a collision energy setting of 70 eV.

Nontargeted LC-MS-based Metabolic Profiling

Metabolic profiling was conducted using an ACQUITY UPLC I-Class system paired with a VION IMS QTof Mass Spectrometer (Waters Corporation, Milford, USA) operating in both ESI positive and negative ion modes. An ACQUITY

UPLC BEH C18 column (1.7 μm, 2.1 × 100 mm) was used for separation in both modes. The mobile phases, water and a 2:3 v/v mixture of acetonitrile and methanol, each containing 0.1% formic acid, were designated as mobile phases A and B, respectively. The elution gradient was set as follows: starting at 1% B, increasing to 30% B over 1 min, 60% B at 2.5 min, 90% B by 6.5 min, reaching 100% B at 8.5 min, held until 10.7 min, then reduced to 1% B at 10.8 min and maintained until 13 min. The flow rate was maintained at 0.4 mL/min and the column temperature at 45°C. Samples were kept at 4°C during analysis, with an injection volume of 1 μL.
Data acquisition was performed in full scan mode covering an m/z range from 50 to 1000, along with MSE mode which includes two independent scans at different collision energies (CE): a low-energy scan at 4 eV and a high-energy scan with a CE ramp from 20 to 45 eV to fragment ions. Argon (99.999% purity) was used as the collision-induced dissociation gas. The scan time was 0.2 s with an interscan delay of 0.02 s. The capillary voltage was set at 2.5 kV, cone voltage at 40 V, source temperature at 115°C, desolvation gas temperature at 450°C, and desolvation gas flow at 900 L/h.

Reverse Transcriptase-Polymerase Chain Reaction (RT-PCR)

Total RNA was extracted using TRIzol Reagent.
Mouse Primers:
Vimentin: forward: 5’- CGGCTGCGAGAGAAATTGC-3’
reverse: 5’- CCACTTTCCGTTCAAGGTCAAG-3’;
PDGFR-α: forward: 5’- GGACTTACCCTGGAGAAGTGAGAA-3’
reverse: 5’- ACACCAGTTTGATGGATGGGA-3’
Ddr2: forward: 5’- CTGTGGGAGACCTTCACCTT-3’
reverse: 5’- TAGATCTGCCTCCCTTGGTC-3’;
Periostin: forward: 5’- ACGGAGCTCAGGGCTGAAGATG-3’
reverse: 5’- GTTTGGGCCCTGATCCCGAC-3’;
Col1: forward: 5’- GCCAAGAAGACATCCCTGAAG-3’;
reverse: 5’- TGTGGCAGATACAGATCAAGC-3’;
Fabp4: forward: 5’- ATGGGGGTGTCCTGGTACAT-3’;
reverse: 5’- CTTTCATGACGCATTCCACCA-3’;
Fabp5: forward: TCTTGTACCCTGGGAGAGAAGT-3’;
reverse: 5’- TTCATGACACACTCCACCACTA-3’;
Cd36: forward: 5’- TCCTGCAGAATACCATTTGATCCT-3’;
reverse: 5’- TGGTTTCTACAAGCTCTGGTTCTTA-3’;
Gapdh: forward: 5’- TGTCGTGGAGTCTACTGGTG-3’
reverse: 5’- ACACCCATCACAAACATGG-3’;
Human Primers:
VIMENTIN: forward: 5’- GGGACCTCTACGAGGAGGAG-3’
reverse: 5’- TCCTCCTGCAATTTCTCCCG-3’;
PERIOSTIN: forward: 5’- CTCATAGTCGTATCAGGGGTCG-3’
reverse: 5’- ACACAGTCGTTTTCTGTCCAC-3’;

DDR2: forward: 5’- CCAGTCAGTGGTCAGAGTCCA-3’
reverse: 5’- GGGTCCCCACCAGAGTGATAA-3’;
PDFGRA: forward: 5’- TGGCAGTACCCCATGTCTGAA-3’
reverse: 5’- CCAAGACCGTCACAAAAAGGC-3’;
FABP4: forward: 5’- ACAGGAAAGTCAAGAGCACCAT-3’
reverse: 5’- TGCGAACTTCAGTCCAGGTC-3’;
GAPDH: forward: 5’- ACAACTTTGGTATCGTGGAAGG-3’
reverse: 5’- GCCATCACGCCACAGTTTC-3’;

Western Blot Analysis

Western blot was performed as described previously. Briefly, proteins from mice or cells were separated by 10% sodium dodecyl sulphate polyacrylamide gel electrophoresis (SDS-PAGE) and then transferred to a polyvinylidene fluoride (PVDF) membrane for 90 min. Skim milk (5%) was used to block the PVDF membranes. Primary antibodies were Vimentin (Cell Signaling, 5741,1:1000), Periostin (R&D, AF2955, 1:1000), GAPDH (Cell Signaling, 5174, 1:2000), p-Akt (Cell Signaling, 4060,1:1000), Akt (Cell Signaling, 9272, 1:1000), GSK-3beta (Cell Signaling, 12456, 1:1000), Fabp4 (Proteintech, 12802-1-AP,1:1000).

Mouse Heart CD34+ ApoE-/- Cell Isolation and Cell Culture

Isolation of CD34-CreER;Rosa26-tdTomato ApoE-/- mouse heart cells was performed as previously described. Then CD34+ cells were sorted with anti-CD34 magnetic beads according to the manufacturer's instructions. The purified CD34+ cells were seeded onto the dishes with 0.1% gelatin and maintained in complete stem cell culture medium, which consists of DMEM, 1% fetal bovine serum, 2% chick embryo extract (MP Biomedical), 100 nM retinoic acid (Sigma-Aldrich), 50 nM 2-mercaptoethanol (Sigma-Aldrich), 2% B27 (Invitrogen), 1% N2 (Invitrogen), 20 ng /ml bFGF (R&D Systems) and 1% P/S. For cell differentiation experiments, CD34+ cells were cultured in plates and maintained in complete stem cell with AngII (concentration gradient: 10μM, 20μM and 50μM; time gradient: 1 days, 3 days and 5 days). For blocking the PPARγ pathway, CD34+ cells were pre-treated with pioglitazone (a PPARγ agonist, 10μM) and GW9662 (a selective PPARγ antagonist, 10μM) for 24 hours and then treated with AngII (50μM, 3 days).

Mouse Serum Enzyme-Linked Immunosorbent Assay (ELISA)

The levels of ANP and BNP in mouse serum were detected by Mouse ANP ELISA kit (Abcam, ab267800) and Mouse BNP ELISA kit (Novus, NBP2-70011) according to the protocol provided.

siRNAs and plasmids Transfection

Cells with>60% sub-confluency were transfected with siRNAs against FABP4 and HA-FABP4 plasmid using Lipofectamine RNAiMAX (Invitrogen). After transfection for 48 hours, relevant experiments were carried out.

Supplementary figures

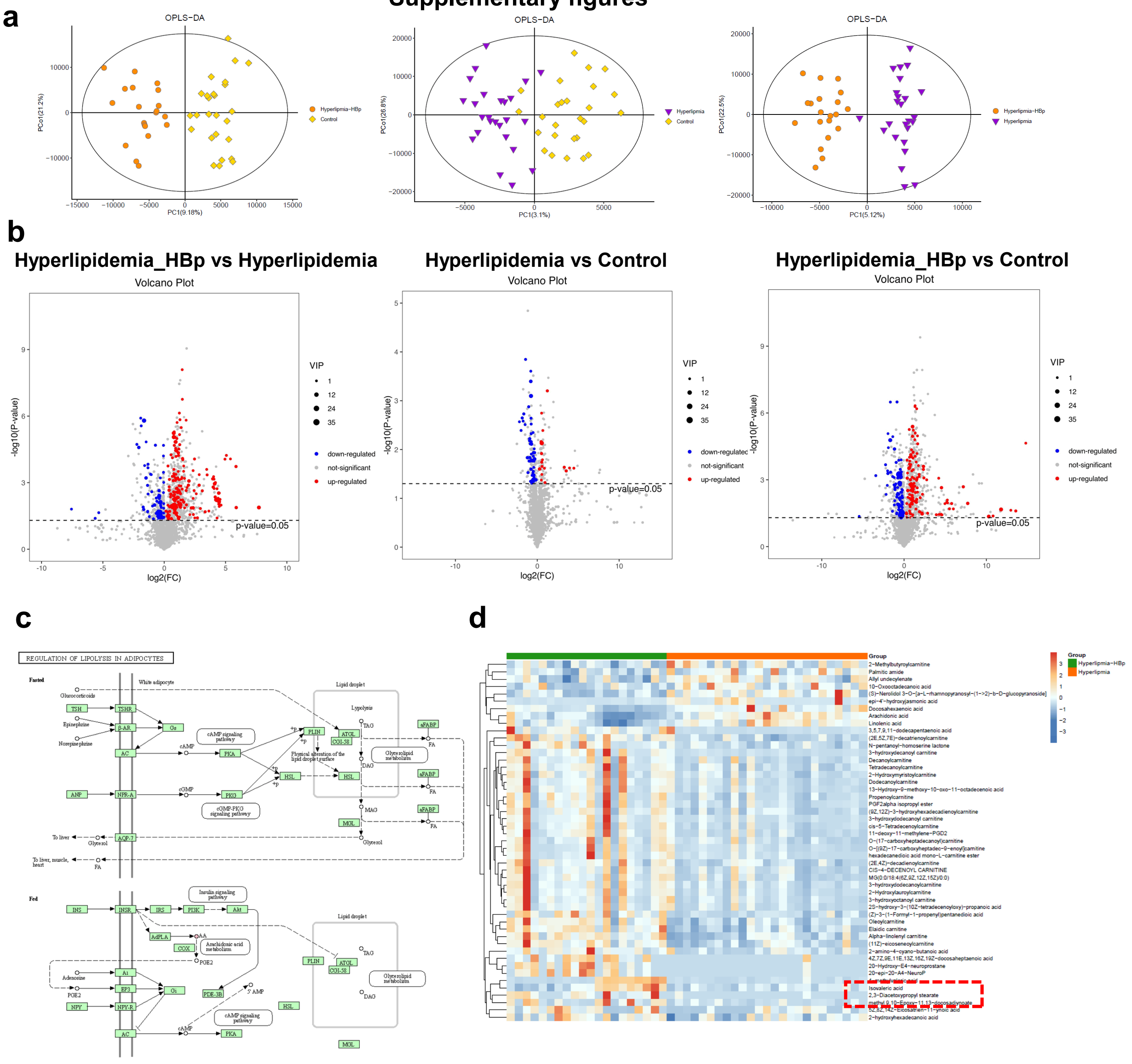

Supplementary Fig.1. a. Analysis of OPLS-DA in different groups of human serum. **b**. Volcanic map of differential metabolites. **c**. KEGG shows the selected metabolic pathways. d. The heat map shows the different metabolites (lipid substance related to fatty acids) between the different groups.

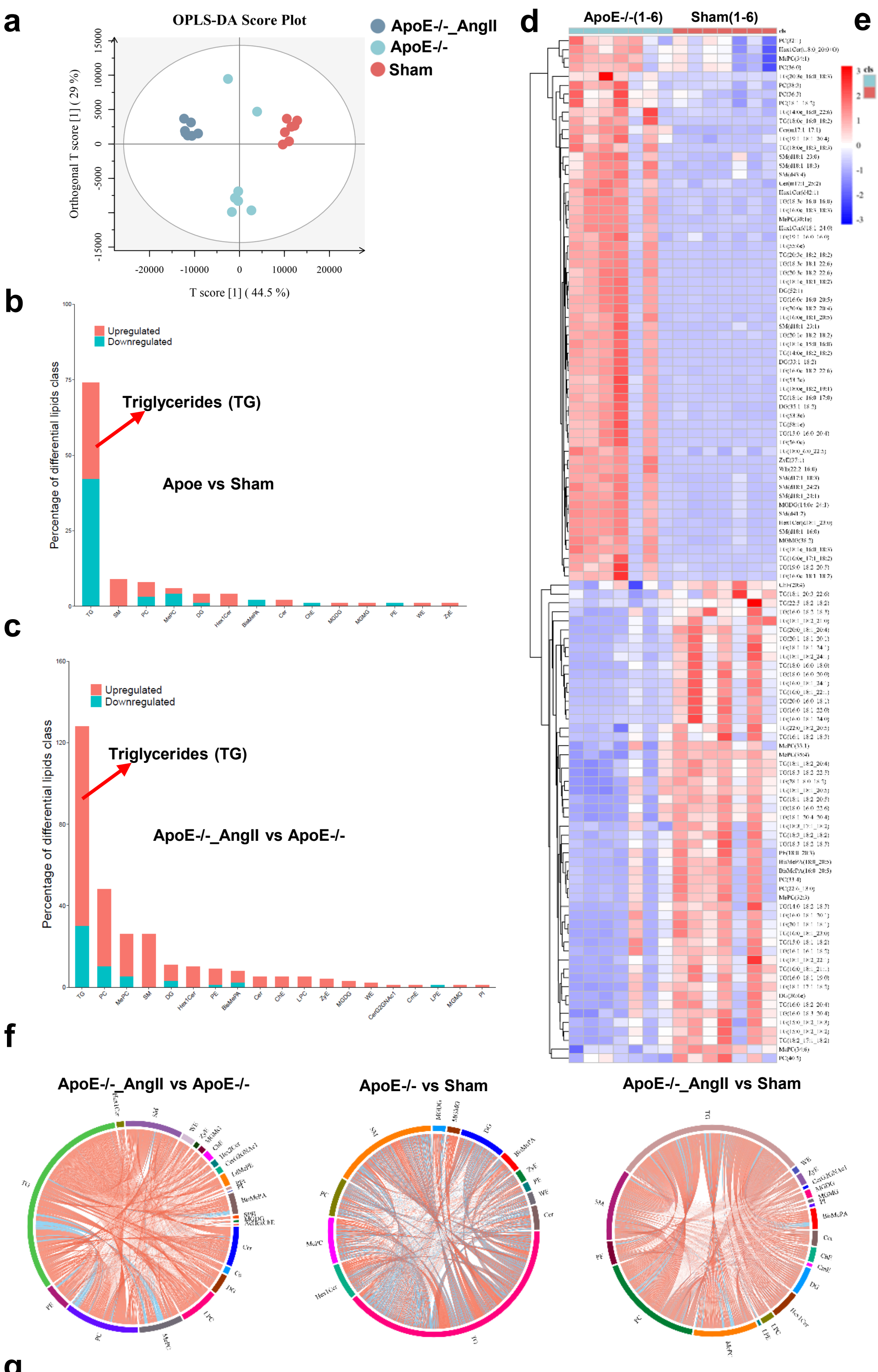

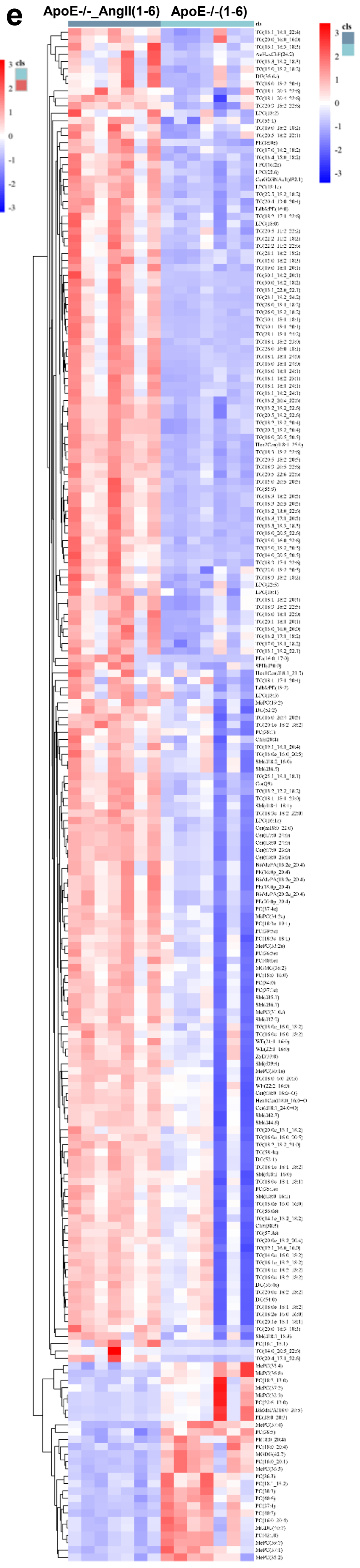

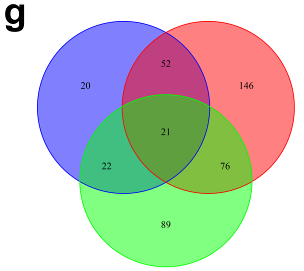

Supplementary Fig.2. a. Analysis of OPLS-DA in different groups of mouse serum. **b-c.** Bar chart of differential lipid class between different group. **d-e.** The heat map shows the different metabolites between the different groups. **f.** Correlation diagram of differential metabolites among different groups. **g.** Venn diagram shows the number of metabolite primes between different groups.

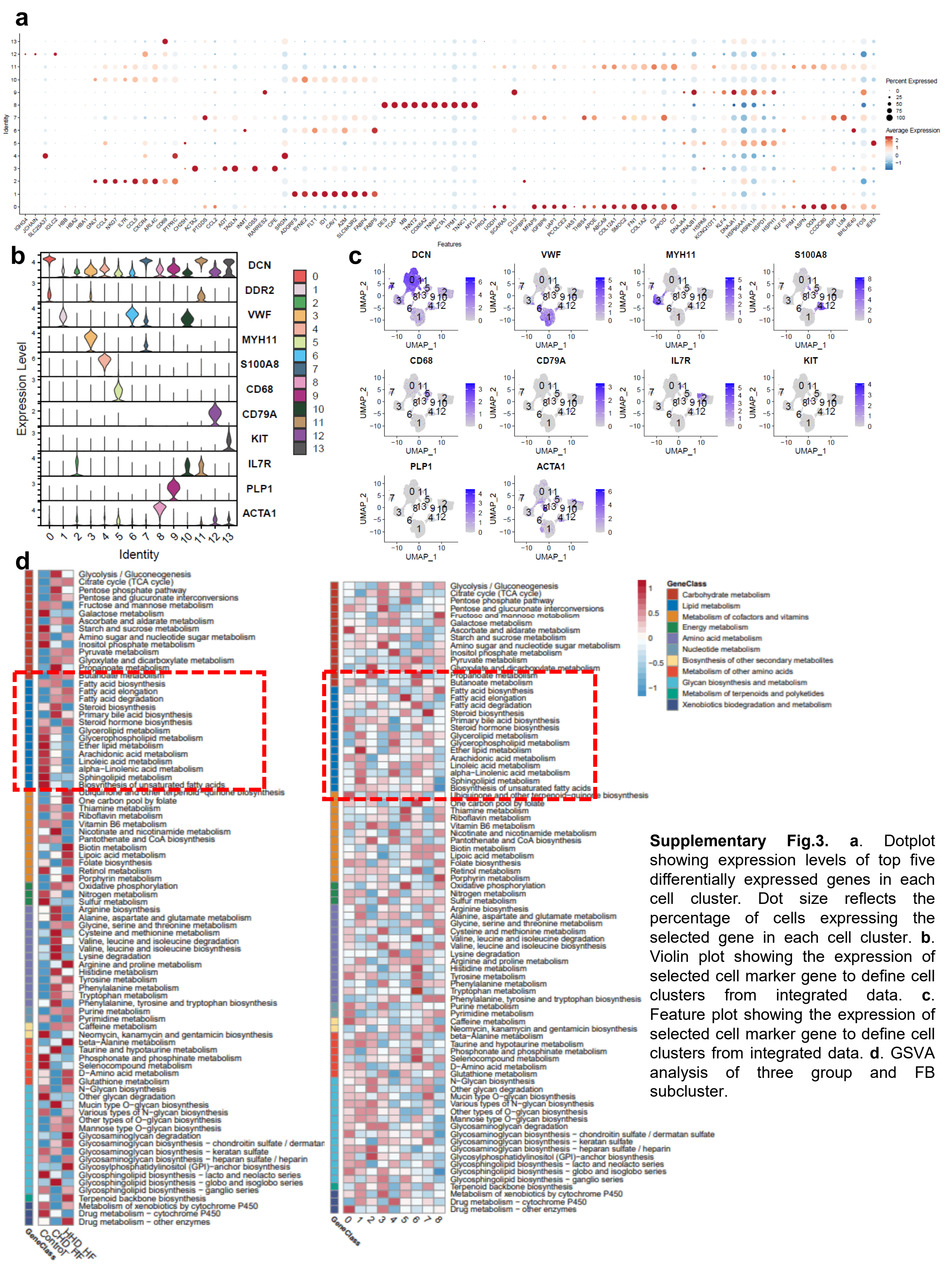

Supplementary Fig.3. a. Dotplot showing expression levels of top five differentially expressed genes in each cell cluster. Dot size reflects the percentage of cells expressing the selected gene in each cell cluster. **b**. Violin plot showing the expression of selected cell marker gene to define cell clusters from integrated data. **c**. Feature plot showing the expression of selected cell marker gene to define cell clusters from integrated data. **d**. GSVA analysis of three group and FB subcluster.

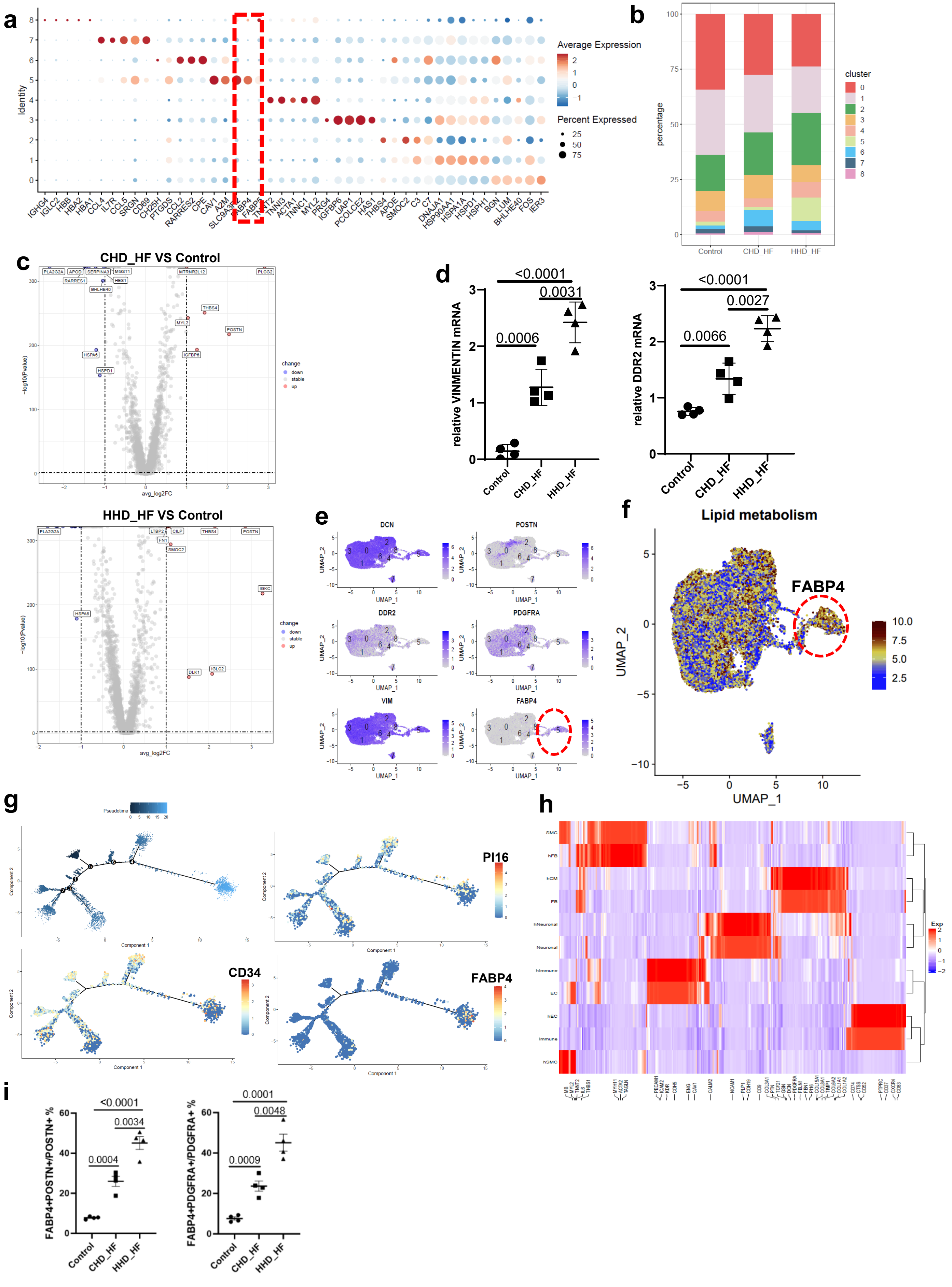
a
Identity
Average Expression
Percent Expressed
25
50
75
IGHG4 IGLC2 HBB HBA2 HBA1 CCL4 IL7R CCL5 SRGN CD69 CH25H PTGDS CCL2 RARRES2 CPE CAV1 A2M SLC9A3R2 FABP4 FABP5 TNNT2 TNNI3 ACTA1 TNNC1 MYL2 PRG4 IGFBP6 UAP1 PCOLCE2 HAS1 THBS4 APOE SMOC2 C3 C7 DNAJA1 HSP90AA1 HSPA1A HSPD1 HSPH1 BGN LUM BHLHE40 FOS IER3
b
percentage
cluster
Control
CHD_HF
HHD_HF
c
CHD_HF VS Control
HHD_HF VS Control
change
down
stable
up
-log10(Pvalue)
avg_log2FC
d
relative VINMENTIN mRNA
relative DDR2 mRNA
<0.0001
0.0031
0.0006
0.0027
0.0066
Control
CHD_HF
HHD_HF
e
DCN
POSTN
DDR2
PDGFRA
VIM
FABP4
UMAP_1
UMAP_2
f
Lipid metabolism
FABP4
UMAP_1
UMAP_2
g
Pseudotime
Component 1
Component 2
PI16
CD34
FABP4
h
SMC
hFB
hCM
FB
hNeuronal
Neuronal
hImmune
EC
hEC
Immune
hSMC
Exp
i
FABP4+POSTN+/POSTN+ %
FABP4+PDGFRA+/PDGFRA+ %
<0.0001
0.0034
0.0004
0.0001
0.0048
0.0009
Control
CHD_HF
HHD_HF

Supplementary Fig.4. a. Dotplot showing the expression of fibroblast genes. Dot size reflects the percentage of cells expressing the selected gene in each cell cluster. **b**. Bar chart showing the percentage of sub-clusters in datasets. **c**. Volcano plot showing the DEGs between datasets (p-value <0.01 and log2FC>1 was labeled). **d.** mRNA levels of fibroblastic markers (VIMENTIN and DDR2) in total fibroblasts isolated from patient heart, GAPDH was used as internal control, Data represent mean±SEM, n=4. **e**. Feature plot showing the expression of selected cell marker gene to define cell clusters from integrated data. **f.** GSVA analysis of three group and FB subcluster. **g.** Analysis of the pseudotime trajectory and expression patterns of CD34, PI16, and FABP4 in the fibroblast. **h.** Correlation analysis of human and mouse cell species. **i.** Graph showing percentage of FABP4+ fibroblast expression in POSTN+, PDGFRA+ fibroblast. Data represent mean ± SEM, n=4.

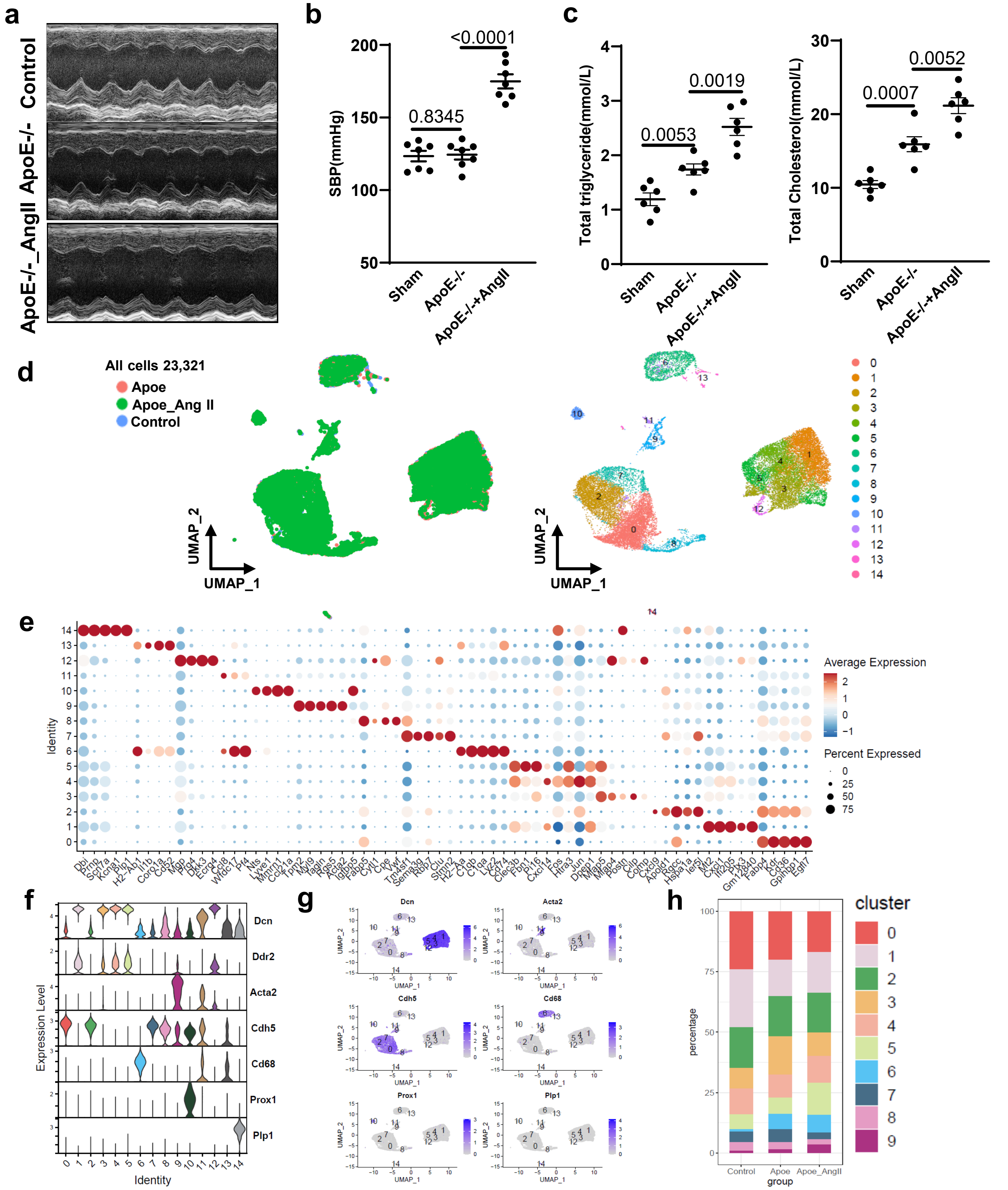

Supplementary Fig.5. a. Representative echocardiography of mice at different stages of pathological cardiac hypertrophy. **b**. Measurement of blood pressure in mouse. Data represent mean±SEM,n=7. **c**. Measurement of total triglyceride and cholesterol in mouse serum. Data represent mean±SEM, n=6. **d.** Umap plot displaying the major cell types and color-coded cell clusters at different stages of pathological cardiac hypertrophy. **e.** Dotplot showing expression levels of top five differentially expressed genes in each cell cluster. Dot size reflects the percentage of cells expressing the selected gene in each cell cluster. **f.** Violin plot showing the expression of selected cell marker gene to define cell clusters from integrated data. **g.** Feature plot showing the expression of selected cell marker gene to define cell clusters from integrated data. **h.** Bar chart showing the percentage of sub-clusters in datasets.

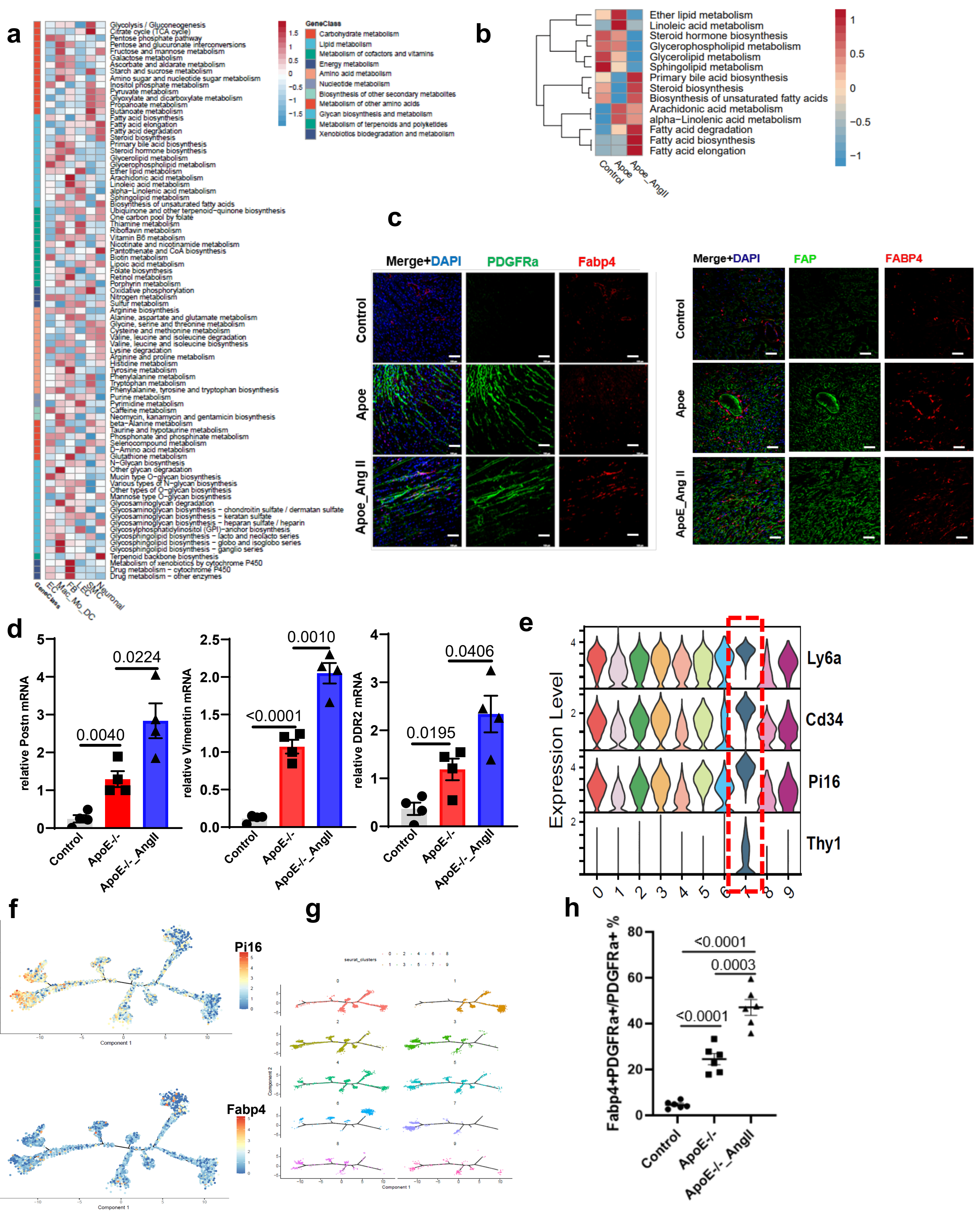

a
Glycolysis / Gluconeogenesis
Citrate cycle (TCA cycle)
Pentose phosphate pathway
Pentose and glucuronate interconversions
Fructose and mannose metabolism
Galactose metabolism
Ascorbate and aldarate metabolism
Starch and sucrose metabolism
Amino sugar and nucleotide sugar metabolism
Inositol phosphate metabolism
Pyruvate metabolism
Glyoxylate and dicarboxylate metabolism
Propanoate metabolism
Butanoate metabolism
Fatty acid biosynthesis
Fatty acid elongation
Fatty acid degradation
Steroid biosynthesis
Primary bile acid biosynthesis
Steroid hormone biosynthesis
Glycerolipid metabolism
Glycerophospholipid metabolism
Ether lipid metabolism
Arachidonic acid metabolism
Linoleic acid metabolism
alpha−Linolenic acid metabolism
Sphingolipid metabolism
Biosynthesis of unsaturated fatty acids
Ubiquinone and other terpenoid−quinone biosynthesis
One carbon pool by folate
Thiamine metabolism
Riboflavin metabolism
Vitamin B6 metabolism
Nicotinate and nicotinamide metabolism
Pantothenate and CoA biosynthesis
Biotin metabolism
Lipoic acid metabolism
Folate biosynthesis
Retinol metabolism
Porphyrin metabolism
Oxidative phosphorylation
Nitrogen metabolism
Sulfur metabolism
Arginine biosynthesis
Alanine, aspartate and glutamate metabolism
Glycine, serine and threonine metabolism
Cysteine and methionine metabolism
Valine, leucine and isoleucine degradation
Valine, leucine and isoleucine biosynthesis
Lysine degradation
Arginine and proline metabolism
Histidine metabolism
Tyrosine metabolism
Phenylalanine metabolism
Tryptophan metabolism
Phenylalanine, tyrosine and tryptophan biosynthesis
Purine metabolism
Pyrimidine metabolism
Caffeine metabolism
Neomycin, kanamycin and gentamicin biosynthesis
beta−Alanine metabolism
Taurine and hypotaurine metabolism
Phosphonate and phosphinate metabolism
Selenocompound metabolism
D−Amino acid metabolism
Glutathione metabolism
N−Glycan biosynthesis
Other glycan degradation
Mucin type O−glycan biosynthesis
Various types of N−glycan biosynthesis
Other types of O−glycan biosynthesis
Mannose type O−glycan biosynthesis
Glycosaminoglycan degradation
Glycosaminoglycan biosynthesis − chondroitin sulfate / dermatan sulfate
Glycosaminoglycan biosynthesis − keratan sulfate
Glycosaminoglycan biosynthesis − heparan sulfate / heparin
Glycosylphosphatidylinositol (GPI)−anchor biosynthesis
Glycosphingolipid biosynthesis − lacto and neolacto series
Glycosphingolipid biosynthesis − globo and isoglobo series
Glycosphingolipid biosynthesis − ganglio series
Terpenoid backbone biosynthesis
Metabolism of xenobiotics by cytochrome P450
Drug metabolism − cytochrome P450
Drug metabolism − other enzymes
GeneClass
EC
Mac_Mo_DC
FB
LEC
SMC
Neuronal
1.5
1
0.5
0
−0.5
−1
−1.5
GeneClass
Carbohydrate metabolism
Lipid metabolism
Metabolism of cofactors and vitamins
Energy metabolism
Amino acid metabolism
Nucleotide metabolism
Biosynthesis of other secondary metabolites
Metabolism of other amino acids
Glycan biosynthesis and metabolism
Metabolism of terpenoids and polyketides
Xenobiotics biodegradation and metabolism
b
Ether lipid metabolism
Linoleic acid metabolism
Steroid hormone biosynthesis
Glycerophospholipid metabolism
Glycerolipid metabolism
Sphingolipid metabolism
Primary bile acid biosynthesis
Steroid biosynthesis
Biosynthesis of unsaturated fatty acids
Arachidonic acid metabolism
alpha−Linolenic acid metabolism
Fatty acid degradation
Fatty acid biosynthesis
Fatty acid elongation
Control
Apoe
Apoe_AngII
1
0.5
0
−0.5
−1
c
Merge+DAPI
PDGFRa
Fabp4
Control
Apoe
Apoe_Ang II
Merge+DAPI
FAP
FABP4
Control
Apoe
ApoE_Ang II
d
relative Postn mRNA
0.0224
0.0040
Control
ApoE-/-
ApoE-/-_AngII
relative Vimentin mRNA
0.0010
<0.0001
Control
ApoE-/-
ApoE-/-_AngII
relative DDR2 mRNA
0.0406
0.0195
Control
ApoE-/-
ApoE-/-_AngII
e
Expression Level
Ly6a
Cd34
Pi16
Thy1
0 1 2 3 4 5 6 7 8 9
f
Pi16
Fabp4
Component 1
Component 2
g
seurat_clusters
Component 1
Component 2
h
Fabp4+PDGFRa+/PDGFRa+ %
<0.0001
0.0003
<0.0001
Control
ApoE-/-
ApoE-/-_AngII

Supplementary Fig.6. a. GSVA analysis of major type of three group. **b**. GSVA analysis of lipid of three group. **c**. Representative images showing specific cell identification by staining with PDGFra, FAP and Fabp4. **d**. mRNA levels of fibroblastic markers and ECM proteins (Postn, Vimentin, DDR2) in total fibroblasts isolated from mouse heart, GAPDH was used as internal control, Data represent mean$\pm$SEM, n=4. **e**. Violin plot showing the expression of selected marker gene of each subclusters. **f.** Pseudotime-dependent expression of Pi16 and Fabp4. **g.** Trajectory analysis of each subclusters of fibroblasts. **h.** Graph showing percentage of FABP4+ fibroblast expression in PDGFRa+ fibroblast. Data represent mean $\pm$ SEM, n=6.

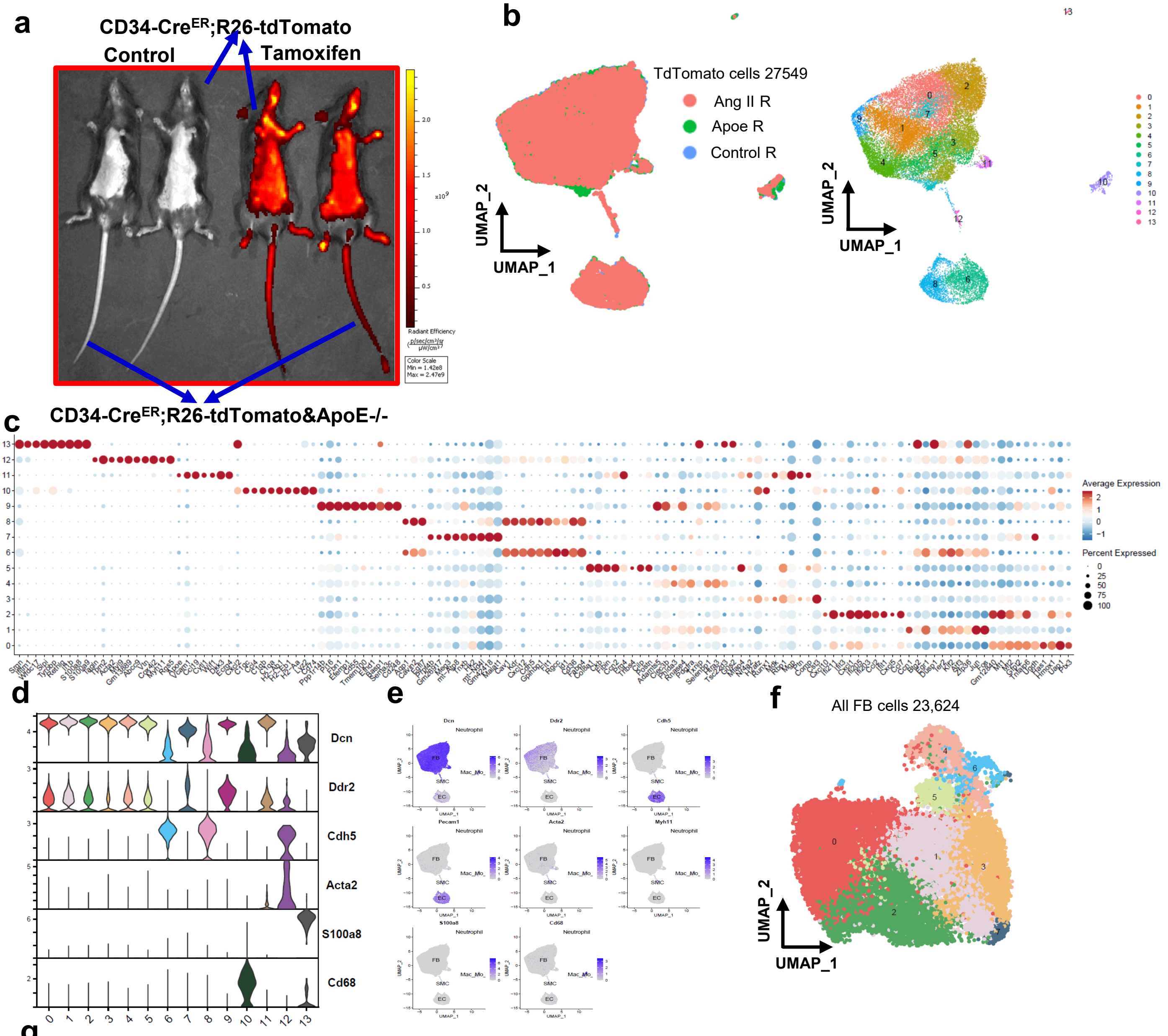

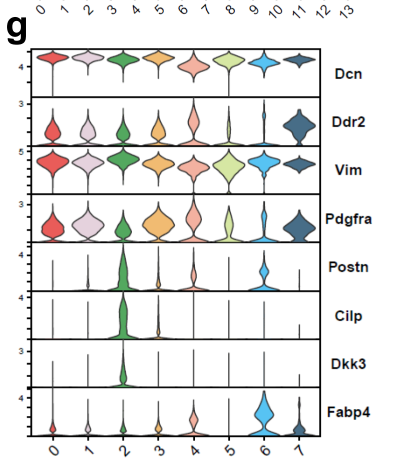

Supplementary Fig.7. a. In vivo imaging of CD34-CreERT2;Rosa26-tdTomato mice with or without tamoxifen induction. **b.** Umap plot displaying the major cell types and color-coded cell clusters at different stages of pathological cardiac hypertrophy. **c.** Dotplot showing expression levels of top five differentially expressed genes in each cell cluster. Dot size reflects the percentage of cells expressing the selected gene in each cell cluster. d. Violin plot showing the expression of selected cell marker gene to define cell clusters from integrated data. e. Feature plot showing the expression of selected cell marker gene to define cell clusters from integrated data. f. Umap plot displaying distribution of fibroblast subpopulations among different datasets after Ang II surgery. g.Violin plot showing the expression of selected marker gene of each subclusters.

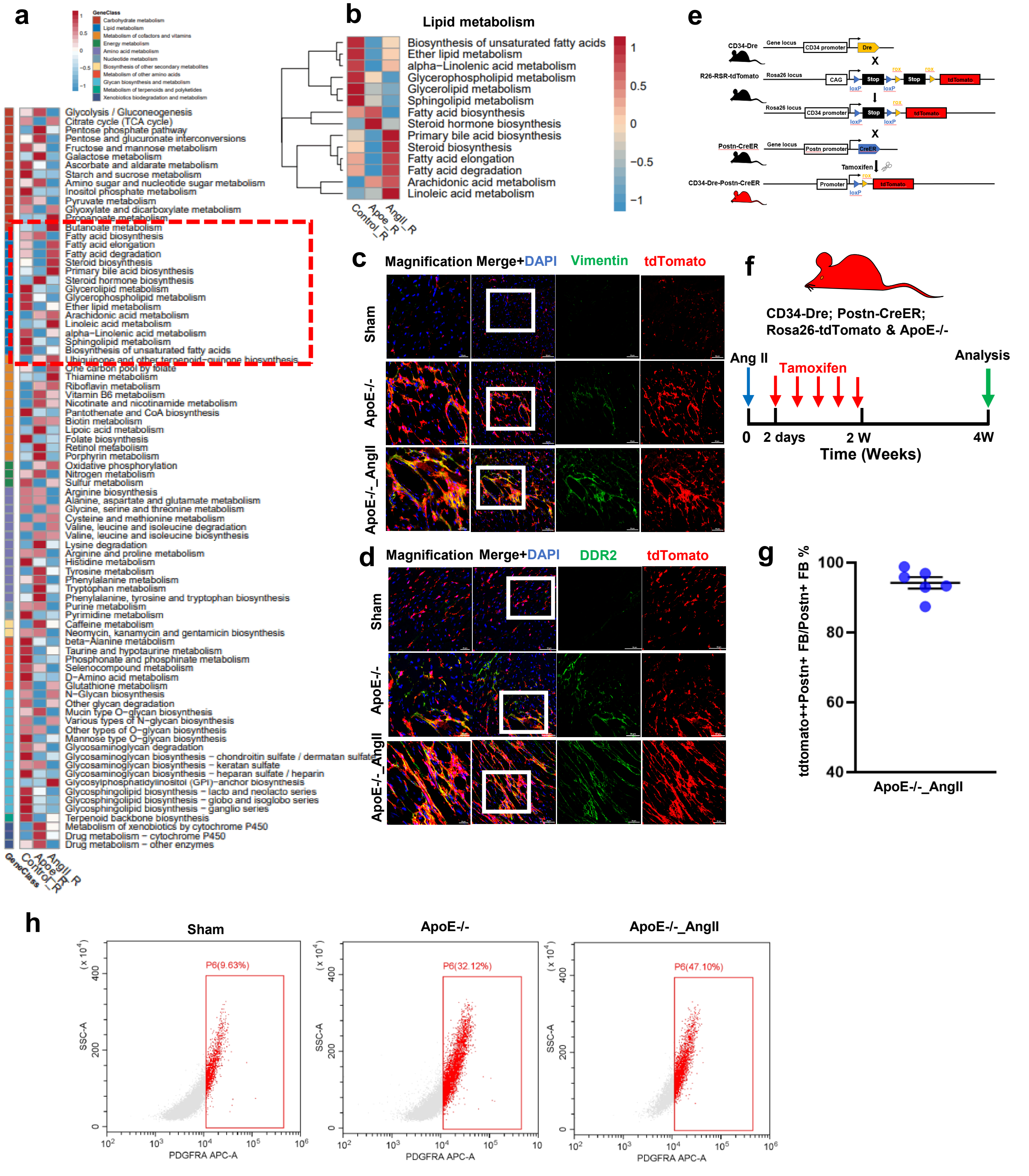

Supplementary Fig.8. **a.** GSVA analysis of major type of three group. **b.** GSVA analysis of lipid of three group. **c-d.** Representative images showing specific cell identification by staining with Ddr2, Vimentin, tdTomato. **e.** The construction of the CD34Dre;PostnCreERT2;R26-RSR-LSL-TdT (Cd34-Dre; Postn-CreER;tdTomato) dual-recombinase-activated lineage tracing mice. **f.** Schematic depicting the pipeline of surgery on CD34-Dre; Postn-CreER;Rosa26-tdTomato & ApoE-/- mouse. **g.** Graph showing percentage of tdtomato+Postn+ fibroblast in Postn+ fibroblast. Data represent mean ± SEM, n=6. **h.** Representative Flow cytometry analysis showing the percentage of tdTomato+PDGFRA+ cell in the heart among three groups.

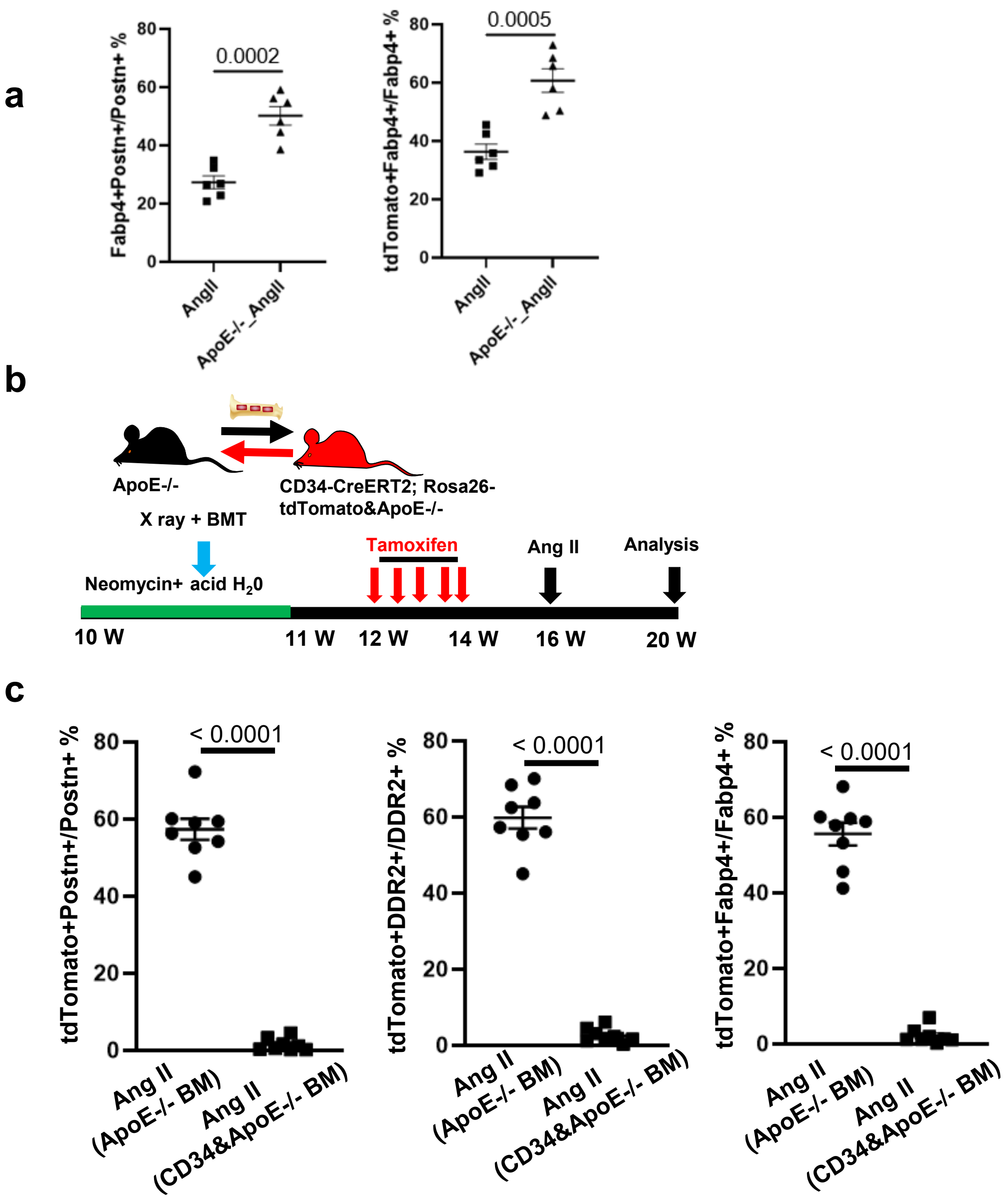

Supplementary Fig.9.

a. Graph showing the percentage of Fabp4+Postn+ and tdtomato+Fabp4+. Data represent mean ± SEM, n=6. **b.**Schematic depicting the pipeline of bone marrow transplantation. Chimeric mice were created by transplanting bone marrow cells from wild-type C57BL/6J mice to CD34-CreERT2;Rosa26-tdTomato (BMT WT TO CD34), or from CD34-CreERT2;Rosa26-tdTomato to WT (BMT CD34 to WT), further treated with tamoxifen and subjected to TAC surgery. **c**. Graph showing percentage of tdTomato expression in vimentin+, DDR2+ fibroblast and Fabp4+ Fibroblast. Data represent mean ± SEM, n=8. BM, bone marrow; BMT, bone marrow transplantation; ApoE-/- BM, bone marrow from ApoE-/-mice; CD34&ApoE-/- BM, bone marrow from CD34-CreERT2;Rosa26-tdTomato&ApoE-/-.

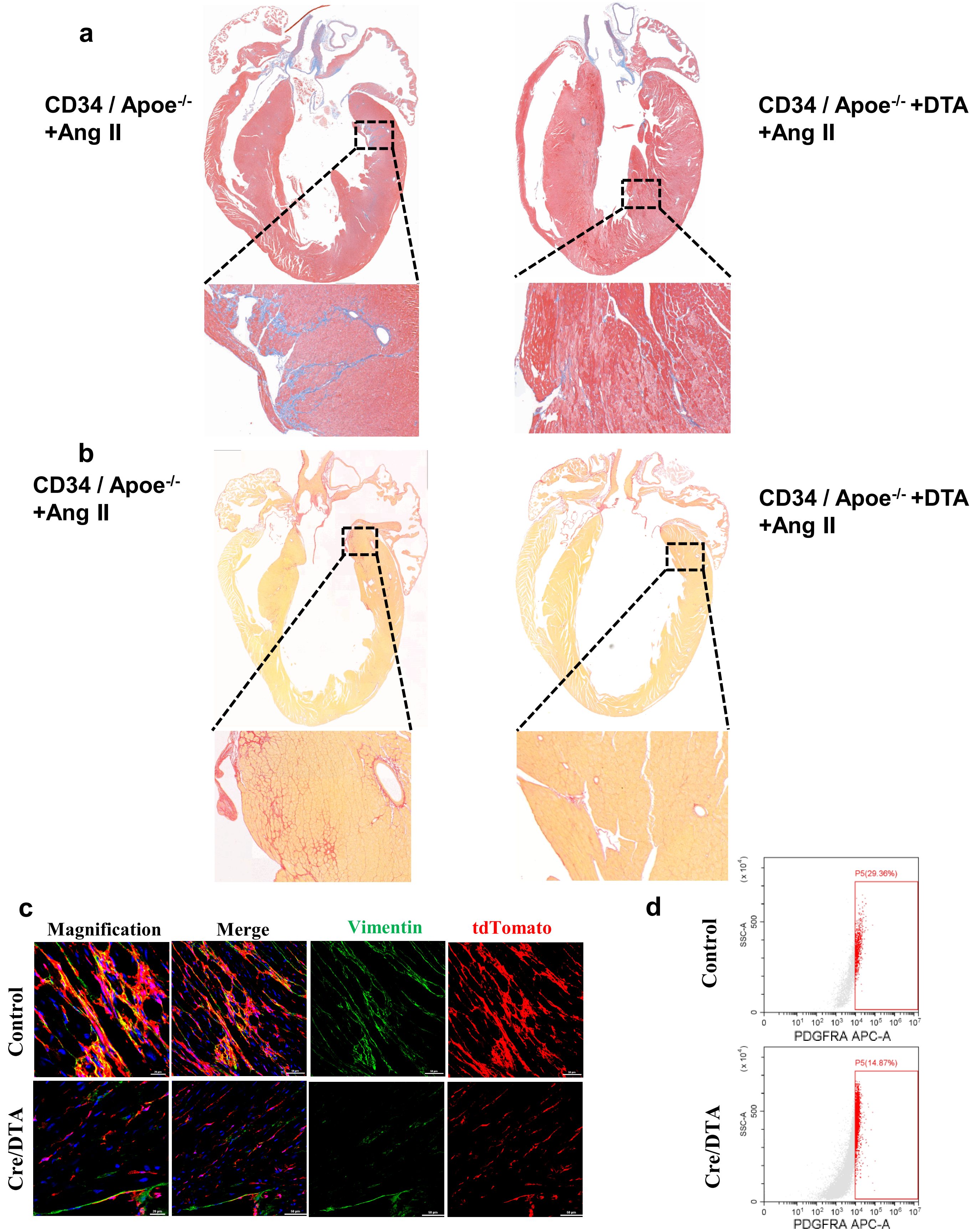

Supplementary Fig.10. a-b. Representative morphological images by Sirius red, Masson staining of hearts 4 weeks after surgery; Sirius red, Masson staining showing different degrees of fibrosis in control group and Cre/DTA group. **c**. Representative immunostaining images showing staining of tdTomato and Vimentin. d. Representative Flow cytometry analysis showing the percentage of tdTomato+PDGFRA+ cell between Control group and Cre/DTA group.

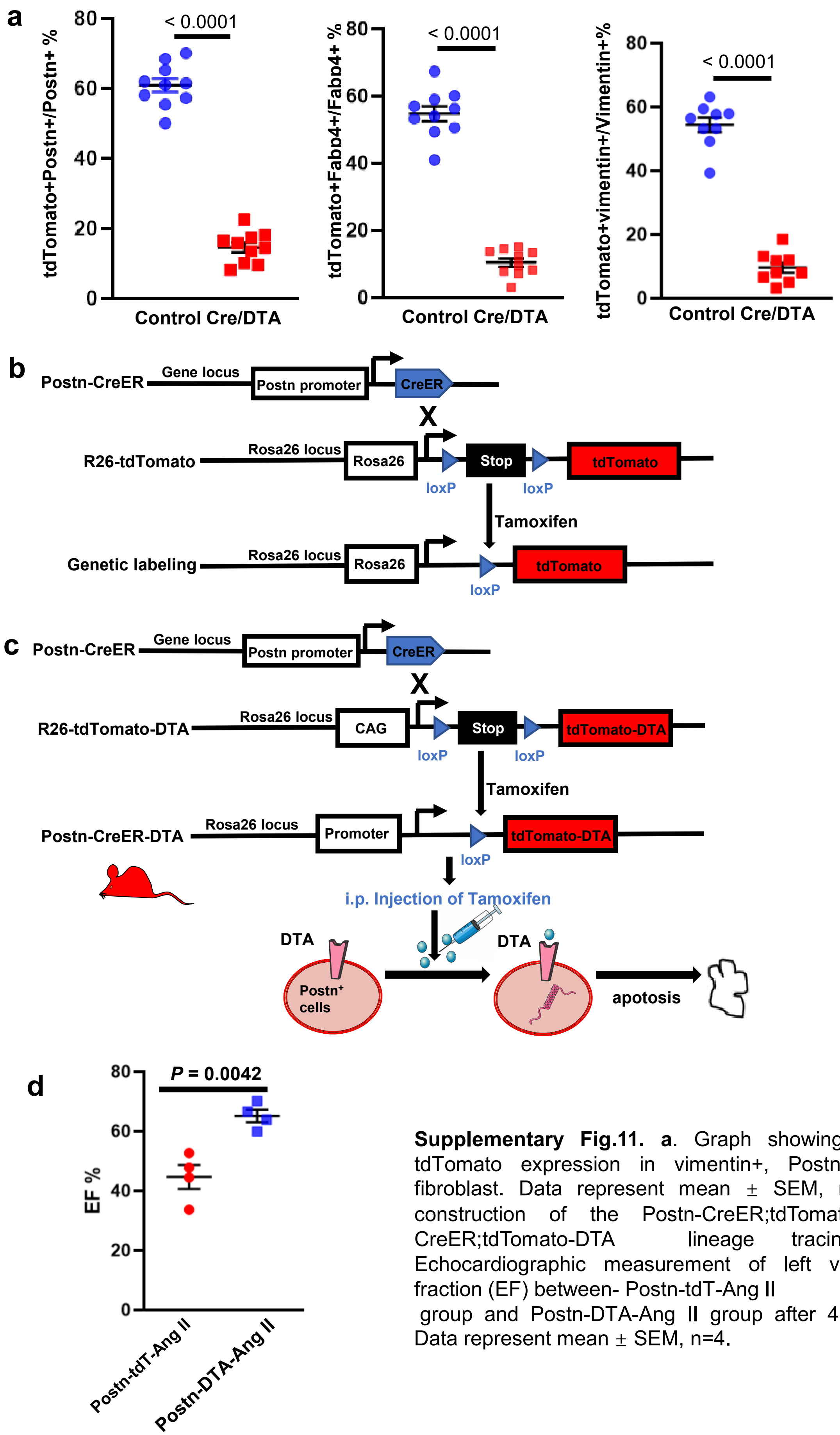

Supplementary Fig.11. **a**. Graph showing percentage of tdTomato expression in vimentin+, Postn+ and Fabp4+ fibroblast. Data represent mean ± SEM, n=10. **b-c.** The construction of the Postn-CreER;tdTomato and Postn-CreER;tdTomato-DTA lineage tracing mice. **d.** Echocardiographic measurement of left ventricle ejection fraction (EF) between- Postn-tdT-Ang II group and Postn-DTA-Ang II group after 4 weeks surgery, Data represent mean ± SEM, n=4.

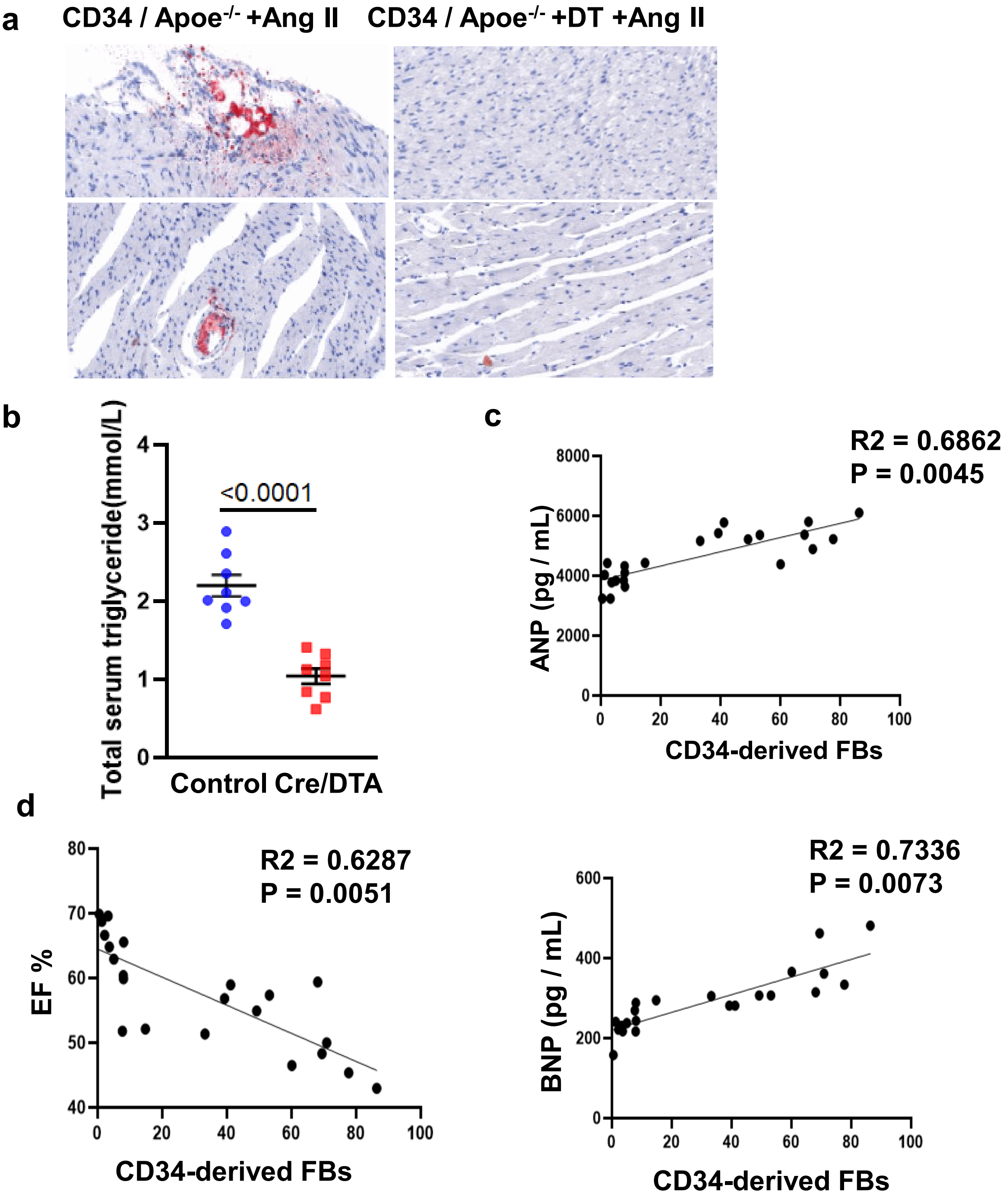

Supplementary Fig.12. a. Oil staining of the heart. **b.** Measurement of total triglycerides (TG) in mouse serum, Data represent mean ± SEM, n=8. **c-d**. Correlation analysis of CD34-derived FBs and EF %, ANP and BNP.

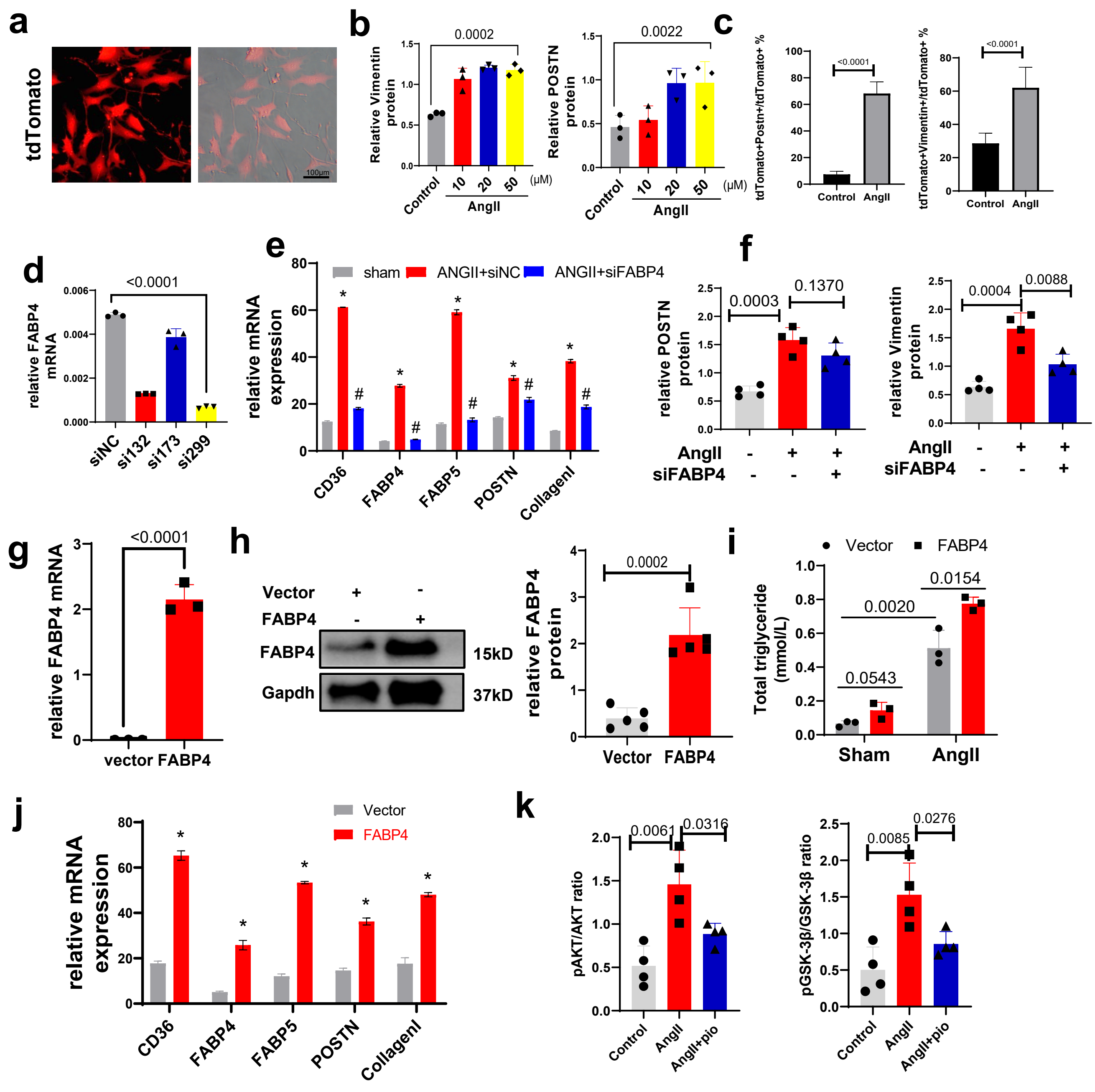

Supplementary Fig.13. a. TdTomato+ cells were isolated from the heart of $ApoE^{-/-}$ 12-week mice (Scale bar=100 μm). **b.** Quantitative data of fibroblastic markers after treated with AngII, n=3. **c.**percentage of postn and vimentin expression in tdTomato+ cells calculated through the immunofluorescence staining. Data represent mean ± SEM, n=5. **d.** FABP4 siRNAs transfection efficiency in CD34+ cells was assessed by qRT-PCR;n=3. **e.** mRNA levels of FABP4, FABP5 ,CD36 and fibroblastic markers, GAPDH was used as internal control, n=6; **f.** Protein expression of FABP4 were determined by western blotting and quantitative data was shown, n=4;**g.** FABP4 vector transfection efficiency in CD34+ cells was assessed by qRT-PCR,n=3; **h.** Protein expression of FABP4 were determined by western blotting and quantitative data was shown, n=5; **i.** Total triglyceride in the cells were assessed, n=3. **j.** mRNA levels of FABP4, FABP5 ,CD36 and fibroblastic markers, GAPDH was used as internal control, n=6; **k.** Quantitative data of pAkt/Akt and p GSK3beta/GSK3beta ratio was shown, Gapdh was used as internal control,n=4.

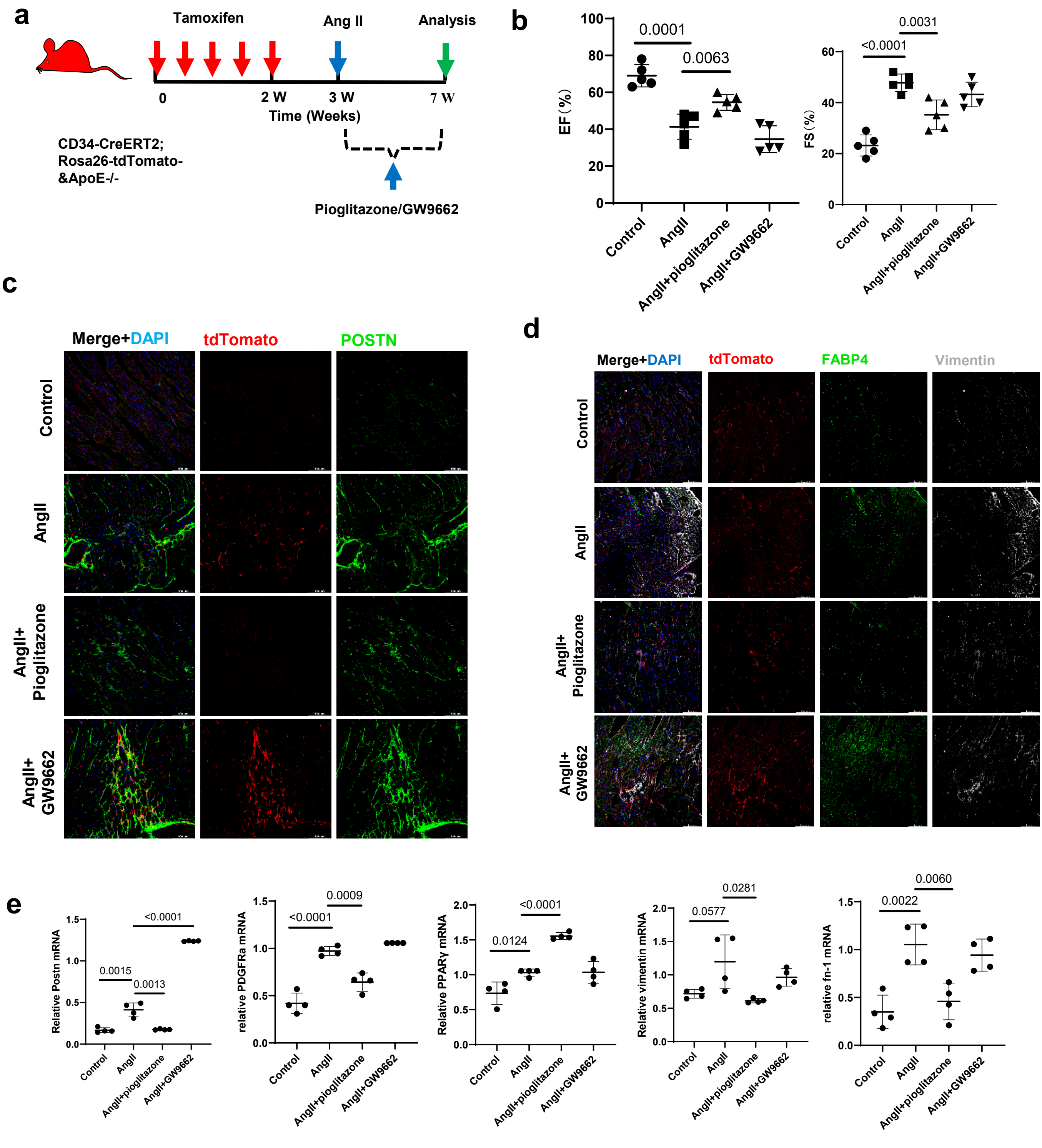

Supplementary Fig.14. a. Experimental scheme of CD34-CreERT2/ApoE mice with hypertension which treated with PPARgama inhibitor or agonist. **b**. Echocardiographic measurement of left ventricle ejection fraction (EF) and fractional shortening (FS) of mice. **c and d**. Representative images showing specific cell identification by staining with POSTN, FABP4, Vimentin and tdTomato. **e**. Graph showing the mRNA levels of Postn , PDGFRa, Pparg and fn-1. Data represent mean ± SEM, n=4.
